# Cell-intrinsic effects of clonal hematopoiesis in heart failure

**DOI:** 10.1038/s44161-023-00322-x

**Published:** 2023-09-04

**Authors:** Wesley T. Abplanalp, Bianca Schuhmacher, Sebastian Cremer, Maximilian Merten, Mariana Shumliakivska, Igor Macinkovic, Andreas M. Zeiher, David John, Stefanie Dimmeler

**Affiliations:** 1 Institute of Cardiovascular Regeneration, Goethe University, Frankfurt, Germany; 2 German Center for Cardiovascular Research DZHK, Partner Site Frankfurt Rhine-Main, Berlin, Germany; 3 Cardiopulmonary Institute, Goethe University, Frankfurt, Germany

**Keywords:** Heart failure, Gene expression profiling, Techniques and instrumentation, Innate immunity, Mutation

## Abstract

Clonal hematopoiesis of indeterminate potential (CHIP) is caused by somatic mutations in hematopoietic stem cells and associates with worse prognosis in patients with heart failure. Patients harboring CHIP mutations show enhanced inflammation. However, whether these signatures are derived from the relatively low number of cells harboring mutations or are indicators of systemic pro-inflammatory activation that is associated with CHIP is unclear. Here we assess the cell-intrinsic effects of CHIP mutant cells in patients with heart failure. Using an improved single-cell sequencing pipeline (MutDetect-Seq), we show that *DNMT3A* mutant monocytes, CD4+ T cells and NK cells exhibit altered gene expression profiles. While monocytes showed increased genes associated with inflammation and phagocytosis, T cells and NK cells present increased activation signatures and effector functions. Increased paracrine signaling pathways are predicted and validated between mutant and wild-type monocytes and T cells, which amplify inflammatory circuits. Altogether, these data provide novel insights into how CHIP might promote a worse prognosis in patients with heart failure.

## Abstract (web summary)

Abplanalp et al. define the transcriptome of cells carrying mutations in a specific gene at a single-cell level (MutDetect-Seq) and apply it to reveal the cell-intrinsic effects of mutations in *DNMT3A* associated with clonal hematopoiesis in patients with heart failure.

## Main

Somatic mutations are acquired during life in our body. Occurrence of leukemogenic mutations in hematopoietic stem cells can result in clonal expansion of affected stem cells, a process called clonal hematopoiesis (CH)^1^. CH is associated with a poor prognosis of affected subjects, which is only in rare cases (0.5–1.0% per year) due to leukemia, but is mediated by other age-related diseases, particularly cardiovascular disease^2,3^. We and others have shown that CH driver mutations in the epigenetic regulators DNMT3A and TET2 are associated with a poor prognosis of patients with heart failure^4,5^. Several studies point to a critical role of inflammatory activation of circulating hematopoietic cells induced by the CH driver mutations^6,7^. Gene editing or silencing of *DNMT3A* and *TET2* in monocytes resulted in increased expression of various inflammatory cytokines^7,8^. Moreover, bone marrow transplantation of *DNMT3A* and *TET2* heterozygous cells impaired heart function after angiotensin II infusion and increased the number of macrophages and T cells infiltrating the heart^7^. In humans, circulating cells of patients with *DNMT3A* CH driver mutations showed an increased expression of inflammatory genes at single-cell level^9^. Overall, these data support a pro-inflammatory effect of CH driver mutation. However, the cell-intrinsic effects of the rather small number of mutated cells, which is between 0.5% and 20% in most patients, are unclear. In this study, we combined single-cell RNA sequencing (scRNA-seq) with Oxford Nanopore long-read sequencing to identify *DNMT3A* mutant cells within the circulating immune cells from patients with heart failure and determined the gene expression at single-cell level.

## Results

### MutDetect-Seq identifies *DNMT3A* mutant cells

**Fig. 1:**
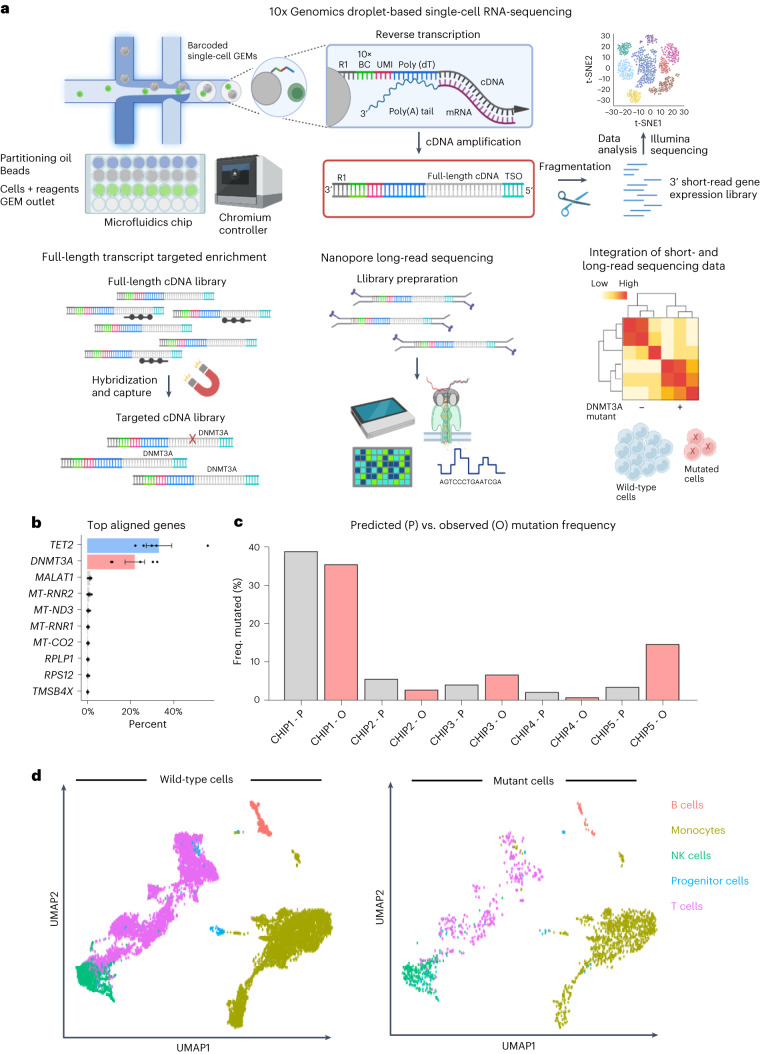
Single-cell targeted long-read sequencing to detect CH somatic mutations in individual cells. **a**, The workflow for targeted long-read sequencing with single-cell resolution is composed of three methodological techniques and bioinformatic data analysis. During scRNA-seq, single cells are partitioned in barcoded gel beads-in-emulsion (GEMs) in which reverse transcription takes place. The first-strand cDNA is purified and amplified, producing the full-length cDNA library. For single-cell 3′ gene expression profiling, a fraction of the cDNA library is fragmented and processed for Illumina short-read sequencing. Additionally, the full-length cDNA library undergoes targeted enrichment by hybridization and capture, followed by Nanopore long-read sequencing. Bioinformatic integration of short-read and long-read sequencing data identifies mutations in targeted transcripts that are linked to gene expression profiles of individual cells. **b**, Exome alignment of long-read sequencing data with top aligned genes and relative on-target percentages (*n* = 5 patients). **c**, Frequency of mutated cells by predicted prior targeted DNA sequencing of *DNMT3A* versus observed mutation frequency in single-cell sequencing analysis. **d**, UMAP of annotated wild-type and mutated single cells with coverage at relative mutation sites. Data show means of each processed library ± s.e.m. (**b**). t-SNE, t-distributed stochastic neighbor embedding.

**Extended Data Fig. 1:**
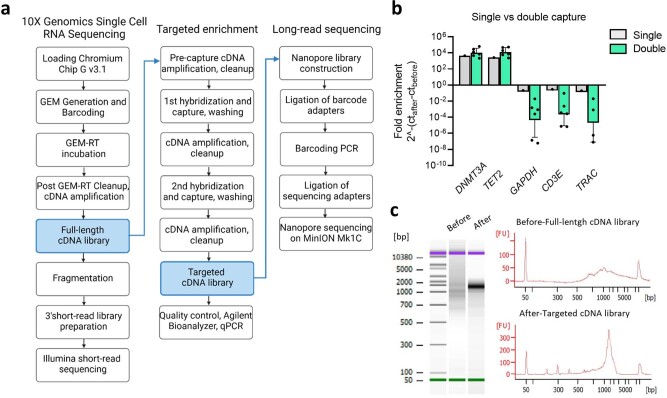
Schematic overview of experimental procedures for single cell targeted long-read sequencing and quality control results. **a**. Detailed workflow of 10X Genomics Single Cell 3‘ gene expression protocol combined with targeted enrichment of full-length transcripts by hybridization and capture and Nanopore long-read sequencing. **b**. Enrichment of targeted transcripts by single and double capture approach (n = 6 patients except for *TRAC* n = 4) relative to cDNA input. The double capture improves the enrichment of targets by a factor of 10 (from 10^3^- to 10^4^-fold) compared to the single capture and more efficiently depletes non-target transcripts. Initial test experiment of single capture (n = 1 patient) is shown. **c**. Representative Agilent Bioanalyzer traces of cDNA libraries before and after targeted enrichment. The amplified full-length cDNA and the post-capture targeted cDNA library were analyzed on an Agilent High Sensitivity DNA Chip. The gel (left) and cDNA profiles (right) suggest the presence of an enriched fraction after two rounds of hybridization and capture compared to the input cDNA library. Panel B: error bars ± SD shown.

To identify *DNMT3A* mutant cells at a single-cell level, we developed MutDetect-Seq (Fig. 1a and Extended Data Fig. 1a). MutDetect-Seq combines short-read single-cell gene expression profiling (10x Genomics) with Oxford Nanopore Technologies (ONT) long-read sequencing. For this purpose, amplified full-length transcripts generated during 10x Genomics library preparation were subjected to two sequential rounds of hybridization and capture for CH gene, using tiled capture probes spanning all exons to capture the full-length transcript (here we used *DNMT3A* and *TET2* as examples; Fig. 1b). This is followed by Oxford Nanopore long-read sequencing of enriched and barcoded transcripts (Fig. 1a and Extended Data Fig. 1a–c). This protocol links the presence of a defined mutation to the 10x barcode of a single cell to compare the gene expression of mutated cells with wild-type cells by bioinformatically integrating the information from short-read and long-read sequencing (Fig. 1a).

**Extended Data Fig. 2:**
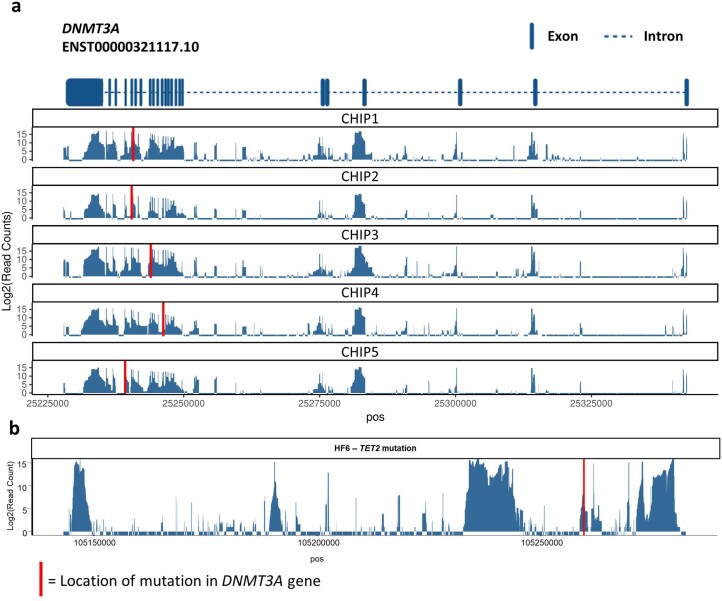
Alignment of long-reads to CHIP gene and mutation locations. **a**. Map of exons and introns for the *DNMT3A* gene (upper panel). Distribution of reads from long read sequencing along the *DNMT3A* gene with non-synonymous mutation location indicated by red vertical line (lower panel). **b**. Validation of detection of somatic mutations in *TET2* for a known carrier by MutDetect-seq.

**Extended Data Fig. 3:**
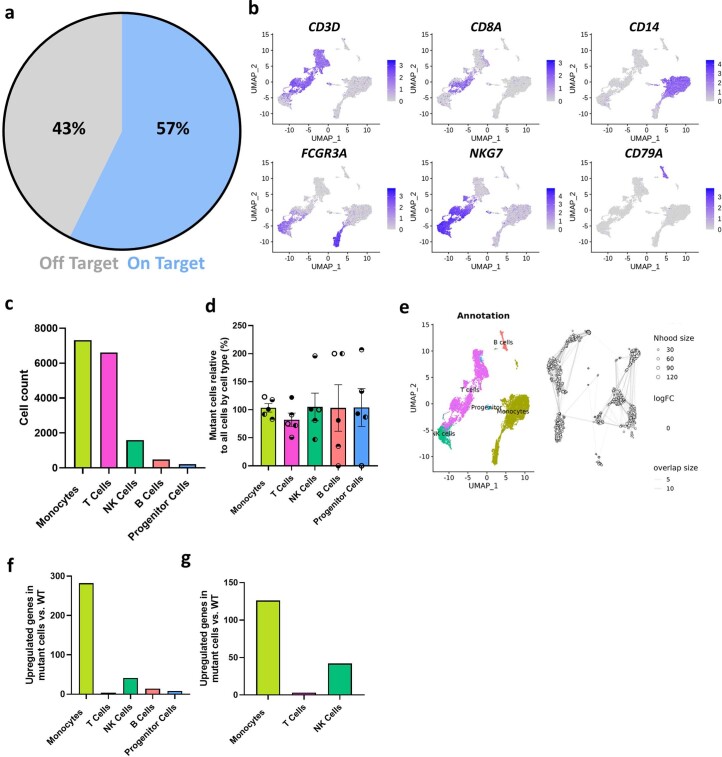
Proportion of cells captured in scRNA-seq and regulated genes. **a**. Proportion of reads mapped to target genes in long read sequencing. **b**. Expression of cell type specific markers shown in Featureplots. **c**. Relative abundance of cells with coverage at patient-respective DNMT3A-mutation sites. **d**. Enrichment plot of mutated cells compared to wild type cell with distribution by class (each dot represents an individual sample, with the fill pattern of the dot representing a specific sample across the different cell types). **e**. Neighborhood distribution analysis by Milo. The Nodes represent the detected neighborhoods by MILO, which are colored by their log fold change between mutated and WT samples. Non-differential abundance neighborhoods (FDR 10%) are colored white, and sizes correspond to the number of cells in a neighborhood. Number of cells shared between adjacent neighborhoods are shown by the graph edges. The node position is set by the position of the neighborhood defining cells in the UMAP representation (left). **f/g**. Number of upregulated genes in DNMT3A mutant cells versus wild type cells by immune cell class (**f**) using all cells and (**g**) when down-sampling to the number of NK cells (that is 1599 cells per cell type). (Panels a-g: n = 5 for relative mutation analysis and pooled differential gene expression analysis. Panel d: data show means of each processed library ± SEM).

MutDetect-Seq was applied to unravel characteristics of *DNMT3A* mutant cells. Specifically, we used circulating immune cells from five patients with heart failure known to have *DNMT3A* mutations (Extended Data Tables 1 and 2)^8^. Targeted enrichment of the cDNA library with *DNMT3A* capture probes resulted in a 10,000-fold mean enrichment of targeted *DNMT3A* transcripts together with a depletion of non-targets, including *GAPDH*, *CD3* and *TRAC* (Extended Data Fig. 1b,c). *DNMT3A* mutations were identified by using an adapted bioinformatic analysis Sicelore pipeline (Extended Data Fig. 1a)^10^. Due to the use of capture probes targeting the full-length transcript, the bioinformatics analysis allowed the identification of specific mutations in the coding sequence of *DNMT3A* (Extended Data Figs. 2a and 3a) and *TET2* (Extended Data Fig. 2b). Focusing on the *DNMT3A* mutations, the resulting mutation matrices of *DNMT3A* clonal hematopoiesis of indeterminate potential (CHIP) carriers were then combined with the single-cell short-read sequencing datasets to identify transcriptomic changes of mutated and non-mutated cells. Overall, we could identify 2,035 *DNMT3A* mutant versus 14,197 wild-type cells.

To assess if our method identifies the predicted number of mutant cells, we compared the percentages of mutant versus wild-type cells identified by MutDetect-Seq with the variant allele frequency (VAF) of the mutations determined by previous targeted DNA sequencing of whole blood. The comparison revealed that the incidence of mutant cells in the individual patients correlated with *DNMT3A* VAFs from previous targeted DNA sequencing (Fig. 1c), suggesting that the mutated cells were correctly identified by the MutDetect-Seq approach, with VAF detection being in a reasonably anticipated range relative to traditional targeted DNA sequencing.

To assess the distribution of mutant cells in the different blood cell populations, total cells were clustered. Dimensional reduction of the total 16,232 cells revealed 13 clusters, which were assigned to monocytes (45%), T cells (41%), natural killer (NK) cells (10%), B cells (3%) and progenitor cells (1%) (Fig. 1d and Extended Data Fig. 3b,c). The annotation and classification of the clusters was determined by expression of cell-type-specific markers (Extended Data Fig. 3b). Consistent with the multipotent pattern of *DNMT3A* CHIP mutations^11^, we detected mutant cells in monocytes, T cells, NK cells, B cells and progenitor cells (Fig. 1d and Extended Data Fig. 3d). No enrichment of mutations was detected in one of the cell types (Extended Data Fig. 3d). Mutant cells did not form an individual cluster but were intermingled with wild-type cells in annotated clusters in uniform manifold approximation and projection (UMAP) plots (Fig. 1d) or when using an advanced clustering tool, such as MILO (Extended Data Fig. 3e).

Next, we determined the effect of *DNMT3A* mutations on gene expression in the individual cell populations. Even after accounting for differing numbers of cells by downsampling of the different cell populations examined, the *DNMT3A* mutation-carrying monocyte population showed the largest number of significantly upregulated genes, whereas the number of significantly upregulated genes was lower in the *DNMT3A* mutant T cells and NK cells (Extended Data Fig. 3f,g; **f** shows results from all cells, and **g** shows results upon downsampling).

### Mutant monocytes have heightened inflammatory signatures

**Fig. 2:**
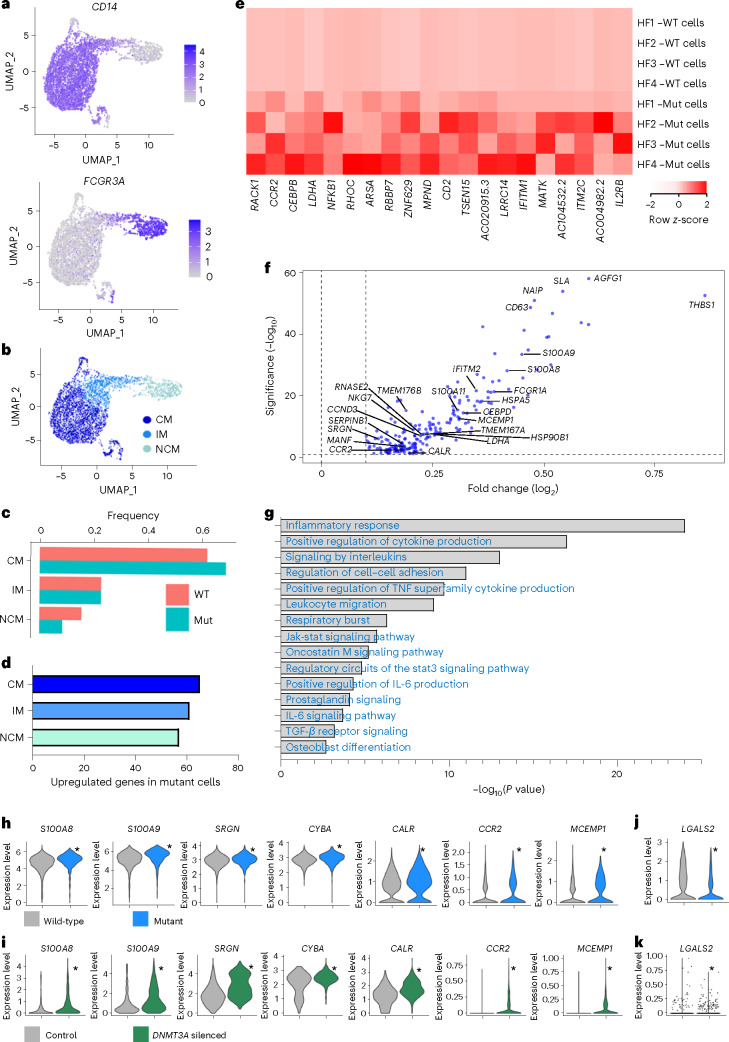
*DNMT3A* mutant monocytes show a pronounced pro-inflammatory phenotype. **a**,**b**, Relative expression of *CD14* and *FCGR3A* (**a**) for identification of monocyte subclasses (shown in **b**). **c**, Distribution of mutated cells compared to wild-type cells by class. **d**, Number of upregulated genes in *DNMT3A* mutant cells versus wild-type cells in different monocyte classes in pooled analysis (downsampled for comparative analysis to NCM cell count, *n* = 1,006 cells per group). **e**, Paired analysis of upregulated genes in mutated versus wild-type cells from monocytes in heat map. **f**,**g**, Upregulated genes in mutant versus wild-type CMs by volcano plot with select significantly called GO terms. **h**,**i**, Select upregulated genes in mutant monocytes (**h**) and validated in vitro by scRNA-seq of THP1 macrophages upon *DNMT3A* silencing (**i**) (*n* = 644 siDNMT3A non-reponsive cells, *n* = 387 siDNMT3A reponsive cells). **j**,**k**, Violin plots of downregulated gene *LGALS2* in mutant monocytes (**j**) and validated in vitro in scRNA-seq of THP1 macrophages (**k**). **a**–**d**,**f**,**g**,**h**,**j**: *n* = 5 for pooled analysis; **e**: *n* = 4 when number of CMs per patient *n* < 5 cells. CM, classical monocyte; IM, intermediate monocyte; Mut, mutant cell; NCM, non-classical monocyte; WT, wild-type cell. **a**,**b**: 7,041 monocytes (total): 4,435 CMs, 1,600 IMs and 1,006 NCMs; **c**: 3,614 WT CMs and 821 mutant CMs; 1,328 WT IMs and 272 mutant IMs; and 906 WT NCMs and 100 mutant NCMs. (Significance was determined for **f**–**k** by two-sided *t*-test, with adjusted *P* < 0.05; in **h**–**k**, significance is denoted by *).

**Extended Data Fig. 4:**
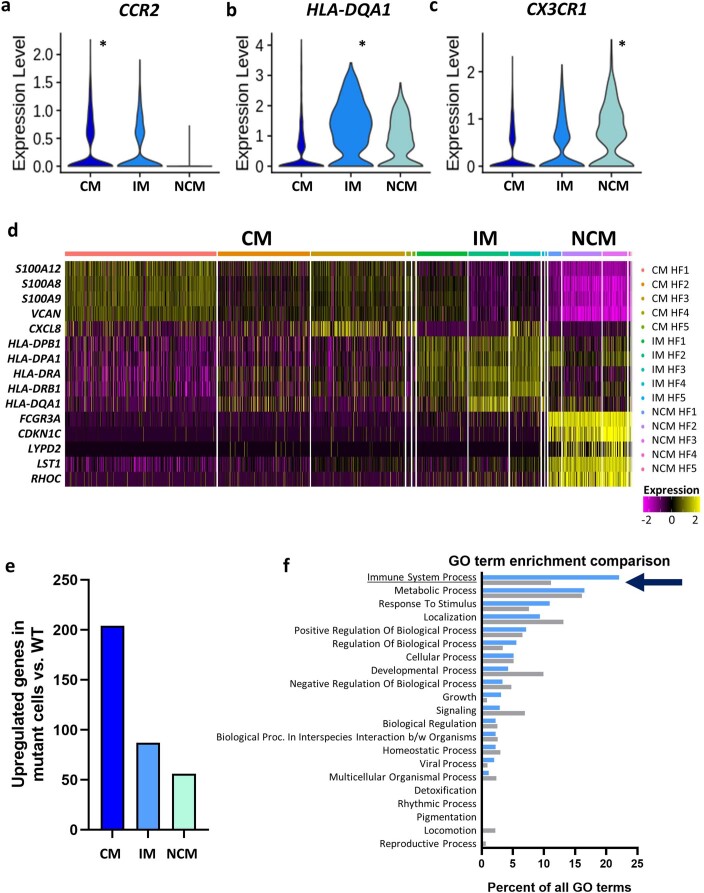
Secondary monocyte class confirmation markers for annotation. **a-c**. Violin plots of common monocyte subclass maker genes for (**a**) classical monocytes, (**b**) intermediate monocytes and (**c**) non-classical monocytes confirming monocyte annotation. **d**. Heatmap of unbiased monocyte subclass maker regulated genes. **e**. Number of upregulated genes in DNMT3A mutant cells versus wild type cells in different monocyte classes in pooled analysis. **f**. Novel DNMT3A-mutant associated gene regulation differentially are called from parent GO term associated with immune processes and inflammation. (CM: classical monocytes; IM: intermediate monocytes; NCM: non-classical monocytes). (Significance denoted by * and reflects adj. p-value < 0.05).

Because *DNMT3A* mutant monocytes showed the highest number of differentially regulated genes (Extended Data Fig. 3f,g), we first performed an in-depth analysis of monocytes. Subclustering of the total monocytes identified classical monocytes (*CD14*^*+*^/*FGCR3A*^*low*^), non-classical monocytes (*CD14*^*low*^/*FGCR3A*^*+*^) and intermediate monocytes (*CD14*^*mid*^/*FCGR3A*^*mid*^) (Fig. 2a,b). Classified monocyte subsets expressed the known characteristic monocyte class marker genes (for example, increased inflammatory markers such as *CCR2* in classical monocytes), thus validating the bioinformatics selection procedure (Extended Data Fig. 4a–d). We first addressed if *DNMT3A* mutant cells might be enriched in one of the subclusters, but we did not find significant changes in proportions between wild-type and mutant cells in the different monocyte classes (Fig. 2c). Because classical monocytes comprise the largest monocyte cell population, we downsampled the three groups to equal numbers to avoid bias. After downsampling, the difference in the number of differentially expressed genes was negligible (Fig. 2d) relative to analysis without downsampling (Extended Data Fig. 4e). Specifically, we detected significant and distinct changes in gene expression patterns when we analyzed mutant and wild-type cells in classical monocytes of the individual patients by a paired comparison (Fig. 2e) and in classical monocytes in a pooled comparison of mutant versus wild-type cells (Fig. 2d,f). The analysis confirmed previous signatures observed when comparing total cells of *DNMT3A* CHIP mutant carriers versus non-CHIP carriers and identified additional pathways along with further enrichment of Gene Ontology (GO) terms related to inflammation (Extended Data Fig. 4f).

**Extended Data Fig. 5:**
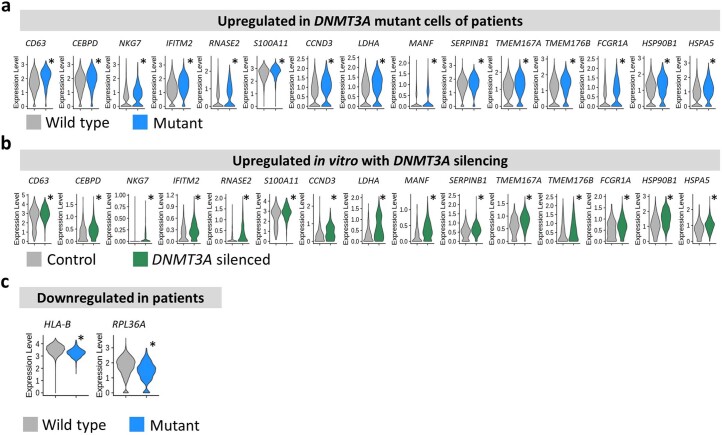
Regulated genes in DNMT3A mutant monocytes and *DNMT3A*-silenced THP1 macrophages. **a**. Violin plots of significantly upregulated genes in mutant vs. wild type cells in monocytes of patients. **b**. Violin plots of significantly upregulated genes in responsive *DNMT3A* silenced THP1 cells. **c**. Violin plots of significantly downregulated genes in mutant vs. wild type cells in monocytes of patients. (Panels **a**/**c**: n = 5 for pooled analysis. Panels **b**: n = 644 siDNMT3A non-reponsive cells, n = 387 siDNMT3A responsive cells. (Significance denoted by * and reflects adj. p-value < 0.05).

In detail, classical monocytes showed differential expression of 204 genes associated with GO terms such as ‘inflammatory response’, ‘positive regulation of cytokine production’ and ‘regulation of cell–cell adhesion’ (Fig. 2g). Unique pathways called in this study also associate with cell killing and PECAM1 interactions. Highly expressed genes in *DNMT3A* mutant monocytes include members of the S100A family, specifically *S100A8* and *S100A9* (Fig. 2h), which are pro-inflammatory, Ca^2+^-binding proteins known to stimulate leukocyte recruitment and induce cytokine secretion^12^. Elevated plasma levels of S100A8/A9 associate with increased risk of coronary events and all-cause mortality in patients with heart failure^13,14^. Notably, *DNMT3A* mutant monocytes further showed enriched expression of *CCR2* (Fig. 2h), which mediates homing of pro-inflammatory bone-marrow-derived cells to the injured heart^15^. Furthermore, the CCAAT/enhancer-binding protein gene subunit delta (*CEBPD*, also known as nuclear factor of IL-6) was upregulated in mutant cells (Extended Data Fig. 5a). CEBPs are induced by LPS, IL-1 and IL-6 and bind to regulatory regions of acute phase and cytokine genes, such as *IL6*, *TNF*, *IL8* and *GCSF*^16^. Interestingly, the proteoglycan serglycin (*SRGN*), which is upregulated in mutant cells (Fig. 2h), has been shown to be involved in TGF-β-mediated migration, macrophage-mediated extracellular matrix changes as well as inflammatory signaling^17–20^. Furthermore, mutant monocytes also show elevated calreticulin (*CALR*) (Fig. 2h). Previous studies showed that activated macrophages secrete calreticulin, which binds to the surface of living target cells and targets their removal by programmed cell phagocytosis^21^.

Interestingly, we also found an upregulation of genes associated with phagocytosis and phagolysosome capacity in *DNMT3A* mutant cells. Thus, the primary component of the microbicidal oxidase system of phagocytes (*CYBA*) is significantly upregulated in mutant cells (Fig. 2h). *CYBA* is part of the phagolysosome machinery, which requires enhanced trafficking and fusion of the phagosome to the lysosome and is supported via *ARF1* and *CD63* (both upregulated in mutant cells) (Fig. 2h and Extended Data Fig. 5a). Excessive monocyte activation and cell targeting may promote poorly directed macrophage phagocytosis having implications for tissue damage after monocyte extravasation. Moreover, CYBA associates with NOX3 to form a functional NADPH oxidase constitutively generating superoxide, which is well established to contribute to progression of heart failure^22,23^.

Downregulated genes include members of the major histocompatibility complex (for example, HLA), genes encoding subunits of the small or large ribosome (for example, *RPL36A*) and the poorly characterized galectin-2 (*LGALS2*) (Fig. 2j and Extended Data Fig. 5c).

As *DNMT3A* driver mutations are loss-of-function mutations, we confirmed *DNMT3A*-dependent gene regulation by silencing *DNMT3A* in THP1 macrophages and analyzed genes of interest by scRNA-seq. We could confirm a significant upregulation of *S100A8/A9*, *SRGN*, *CYBA*, *CALR*, *CCR2* and *MCEMP1* as well as a decrease in *LGALS2* in *DNMT3A*-silenced THP1 macrophages (Fig. 2i,k and Extended Data Fig. 5b). Together, these data decipher the specific pro-inflammatory gene activation in *DNMT3A* mutant monocytes, demonstrating additional molecules, such as proteoglycan serglycin, phagolysosome genes, *MCEMP1* and calreticulin, previously undescribed for CHIP-carrying patients with heart failure.

### Dysregulated gene expression in *DNMT3A* mutant-carrying T cells

**Fig. 3:**
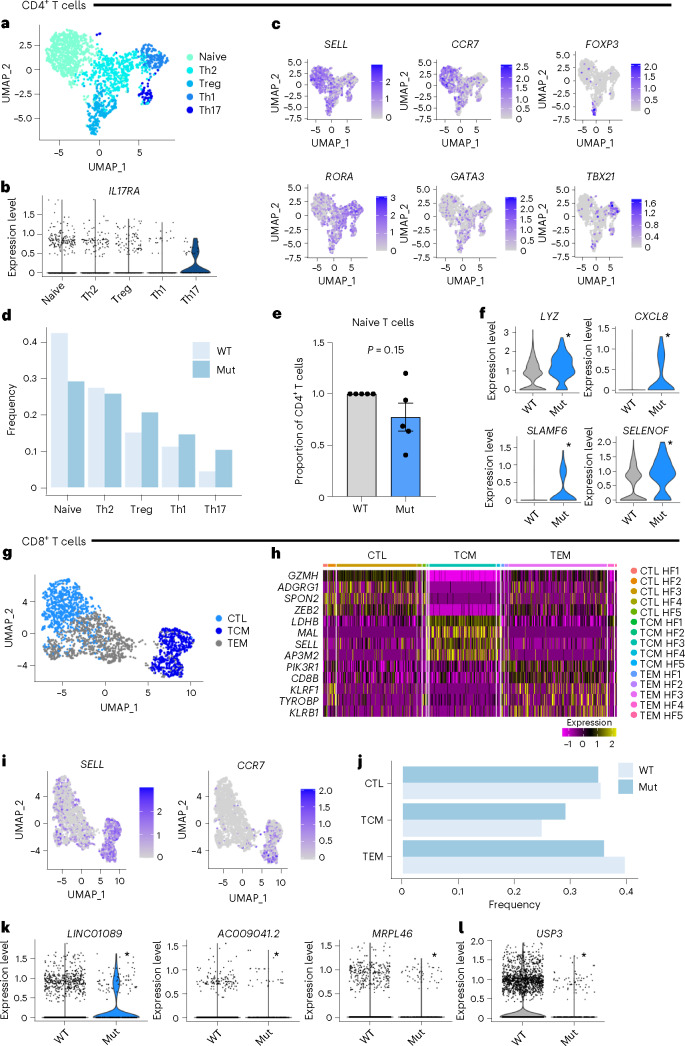
Role of DNMT3A mutations in CD4^+^ and CD8^+^ T cells. **a**, UMAP of CD4^+^ T cells with coverage at mutation site locations. **b**, Violin plot of *Il17RA* in CD4^+^ T cell subsets. **c**, Feature plots showing relative expression of activation markers and T helper subset markers. **d**, Relative abundance of T cell subsets by mutation status. **e**, Proportion of CD4^+^ naive T cells in wild-type and mutant cells by patient. **f**, Upregulated genes in mutant CD4^+^ T cells. **g**, UMAP of CD8^+^ T cells with coverage at the mutation site. **h**, Heat map of genes identifying CD8^+^ T cell subsets. **i**, Feature plots showing relative expression of activation markers. **j**, Relative abundance of CD8^+^ T cell subsets by mutation status. **k**,**l**, Genes upregulated (**k**) and downregulated (**l**) in CD8^+^ T cells. **a**–**d**,**f**: *n* = 2258 wild-type cells and *n* = 125 mutant cells. **e**: *n* = 887 wild-type cells and *n* = 34 mutant cells. **g**–**l**: *n* = 3,469 wild-type cells and *n* = 289 mutant cells. **e**: *P* value was determined by two sided *t*-test. Significance was determined for **f**,**k**,**l** by two-sided *t*-test, with adjusted *P* < 0.05, with significance denoted by *. For **e**: data show means of each patient sample ± s.e.m. CTL, cytotoxic like; Mut, mutant; TCM, T central memory; TEM, T effector memory; WT, wild-type.

Although we detected a low number of significantly upregulated genes in T cells, we wanted to explore the influence of *DNMT3A* mutations on T cell subsets in these smaller populations. Subclustering of annotated T cells revealed CD4^+^ and CD8^+^ T cells and their subpopulations identified by typical markers (Fig. 3a–c,g–i). Patient-specific analysis was only powered to assess naive T cells, with grouped analysis performed for other populations. These data do not demonstrate a significant decrease in the proportion of mutant cells in naive CD4^+^ cell population or increases in effector cell populations (Treg, Th1, Th2 or Th17) (Fig. 3d,e). However, grouped analysis demonstrated 21 upregulated genes in CD4^+^ mutant cells versus wild-type cells, which confirmed a relative induction of effector function in mutant T cells (Fig. 3f)^24–28^. Thus, established markers of T cell effector and killing function, such as lysozyme (*LYZ*) and *CXCL8*, are significantly upregulated in mutant CD4^+^ T cells. In addition, CD4^+^ mutant T cells showed increased expression of drivers of proliferation, such as selenoprotein F (*SELENOF*), and markers of T cell differentiation, such as *SLAMF6* (Fig. 3f).

When analyzing the second major T cell cluster, CD8^+^ T cells (Fig. 3g–i), we found no notable change in cluster distribution (Fig. 3j). Surprisingly, we found only three significantly upregulated genes (Fig. 3k) and one downregulated gene (Fig. 3l) in CD8^+^ T cells, despite CD8^+^ T cells being more abundant relative to CD4^+^ T cells (289 mutant CD8^+^ T cells (7.7% of CD8^+^ population) and 125 mutant CD4^+^ T cells (5% of CD4^+^ population)). The upregulated genes are poorly described, with one RNA having a non-coding status. Interestingly, the downregulated gene USP3 is a ubiquitin ligase, which is known to inhibit type I interferon signaling by deubiquitinating RIG-I-like receptors specifically during viral infection^29^.

Given that NK cells displayed the second highest number of differentially upregulated genes in the peripheral blood mononuclear cell (PBMC) population (41 genes), we finally assessed the impact of CHIP mutations on these cells. Interestingly, NK cells showed upregulation of the important lysozyme molecule (*LYZ*) by 1.4-fold (adjusted *P* = 3.9 × 10^−16^). Neutrophils use lysozyme for selected killing via apoptosis^30^.

Together, our findings suggest a predominant impact of *DNMT3A* mutations on CD4^+^ T cells and NK cells, whereas CD8^+^ T cells appear less affected.

### *DNMT3A* silencing alters CD4^+^ T cell and NK cell activation

**Fig. 4:**
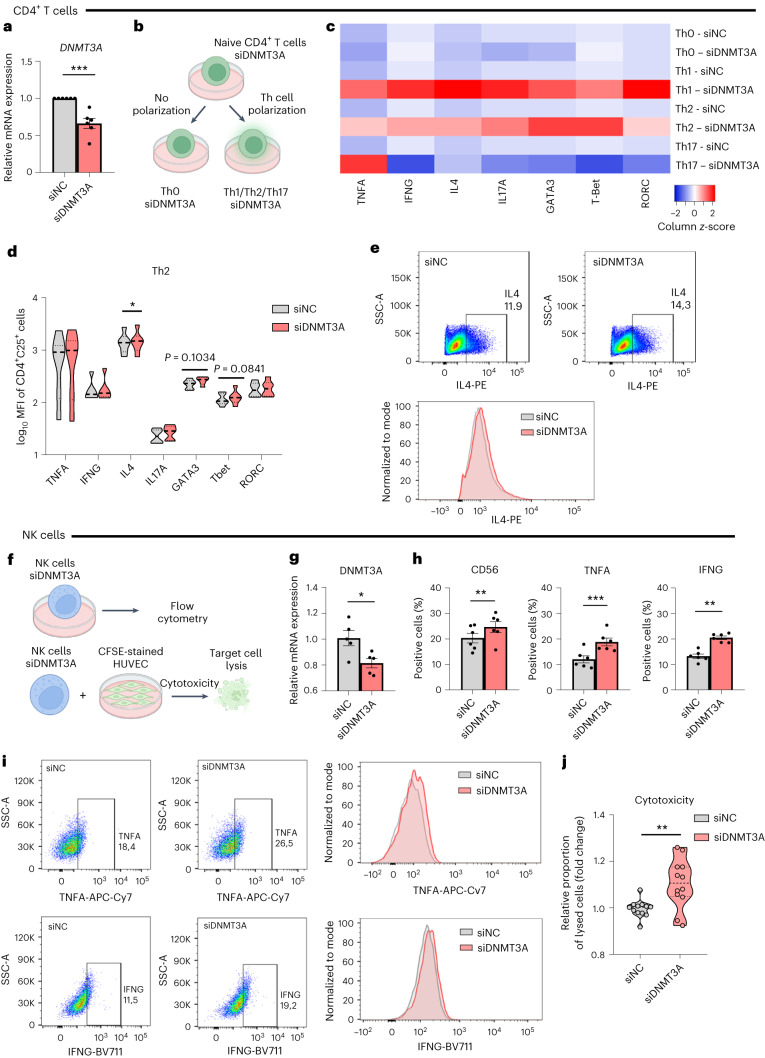
DNMT3A silencing promotes an effector phenotype of CD4^+^ T cells and NK cells. **a**. *DNMT3A* expression level after siRNA silencing in CD4^+^ T cells (*n* = 6 donors) 3 d after siRNA-mediated silencing, *P* = 0.0005. **b**, Experimental procedure to analyze DNMT3A in CD4^+^ T cells. **c**, Heat map showing relative induction of activation markers in Th0 and Th1/Th2/Th17-polarized CD4^+^ T cells after *DNMT3A* silencing (*n* = 6 donors) relative to donor-matched siRNA control (siNC) values. **d**, Mean (geometric) fluorescence intensity (MFI) values of activation markers in *DNMT3A*-silenced and Th2-polarized CD4^+^ T cells (*n* = 6 donors), *P* = 0.0463 for IL-4. **e**, Representative flow cytometry plots (top) and histograms (bottom) of IL-4 in *DNMT3A*-silenced and Th2-polarized CD4^+^ T cells. **f**, Scheme of NK cell readouts to address effects of *DNMT3A* silencing. **g**, *DNMT3A* expression level after siRNA silencing in NKL cells (*n* = 5 biologically independent samples, two independent experiments), *P* = 0.0232. **h**, Flow cytometry analysis of CD56, TNFA and IFNG in *DNMT3A*-silenced NKL cells (*n* = 6 biologically independent samples, three independent experiments), *P* = 0.0032, 0.0009 and 0.0029 for CD56, TNFA and IFNG, respectively. **i**, Representative flow cytometry plots (left) and histograms (right) of TNFA and IFNG in *DNMT3A*-silenced NKL cells. **j**, Fold change of 7-ADD^+^ HUVECs after co-culture with *DNMT3A*-silenced NKL cells compared to siNC NKL (*n* = 12 biologically independent samples, four independent experiments), *P* = 0.0028. Data show means ± s.e.m. (**a**,**g**,**h**) or medians (**c**,**d**). Statistical significance was assessed by two-tailed unpaired (**a**,**g**,**j**) or paired (**d**,**h**) *t*-test (*P < 0.05, **P < 0.01, ***P < 0.001). Source data

**Extended Data Fig. 6:**
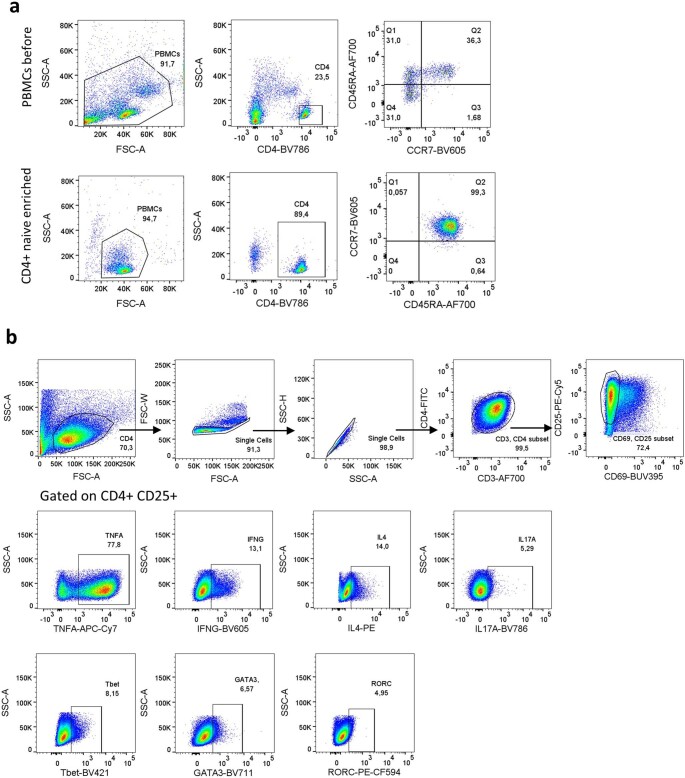
Naive CD4 + T cell isolation and T cell gating strategies. **a**. Enrichment of naive CD4 + T (lower panel) cells from PBMCs (upper panel). Naive CD4+ T cells express CD45RA and CCR7. **b**. Identification of T helper cell markers in CD4 + CD25+ T cells differentiated from naive CD4+ T cells. Arrows indicate sequential gates.

To assess if *DNMT3A* indeed directly affects T cell functions, we silenced *DNMT3A* in naive CD4^+^ T cells (Fig. 4a and Extended Data Fig. 6a) and performed in vitro activation and polarization into Th1, Th2 and Th17 CD4^+^ T cell subsets (Fig. 4b,c). Whereas non-polarized CD4^+^ T cells showed little response to *DNMT3A* silencing (Fig. 4c, Th0 cells, and Extended Data Fig. 6b), T helper cell cytokines and transcription factors in *DNMT3A*-silenced CD4^+^ T cells showed a global activation profile when exposed to Th1-polarizing and Th2-polarizing conditions with significantly increased levels of IL-4 in Th2 cells (Fig. 4c–e and Extended Data Fig. 6b). These data suggest that Th1 and Th2 CD4^+^ T cells may gain effector functions in response to *DNMT3A* silencing.

**Extended Data Fig. 7:**
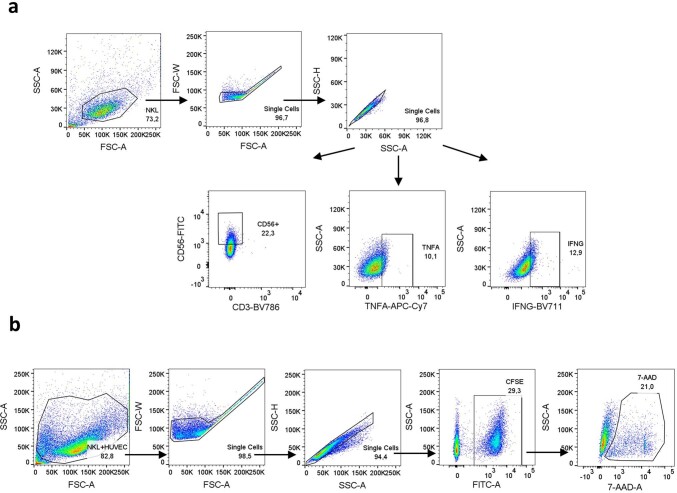
NK cell gating strategies. **a**. Detection of CD56, TNFA and IFNG in NKL cells. **b**. NK cell cytotoxicity assay. CFSE-stained HUVEC lysed by NK cells are identified as CFSE + 7-ADD+ cells. Arrows indicate sequential gates.

To investigate the role of *DNMT3A* in NK cells, *DNMT3A* was silenced in the NK cell line NKL (Fig. 4f,g). *DNMT3A*-silenced NK cells showed upregulation of the NK cell marker CD56 as well as increased levels of the effector cytokines *TNFA* and *IFNG* (Fig. 4h,i and Extended Data Fig. 7a). In line with this observation, *DNMT3A*-silenced NK cells showed increased cytotoxic capacity toward co-cultured human umbilical venous endothelial cells (HUVECs) (Fig. 4j and Extended Data Fig. 7b).

These data support a direct effect of *DNMT3A* on functional properties of CD4^+^ T cells and NK cells.

### Mutant to wild-type cell interactions augment inflammation

**Fig. 5:**
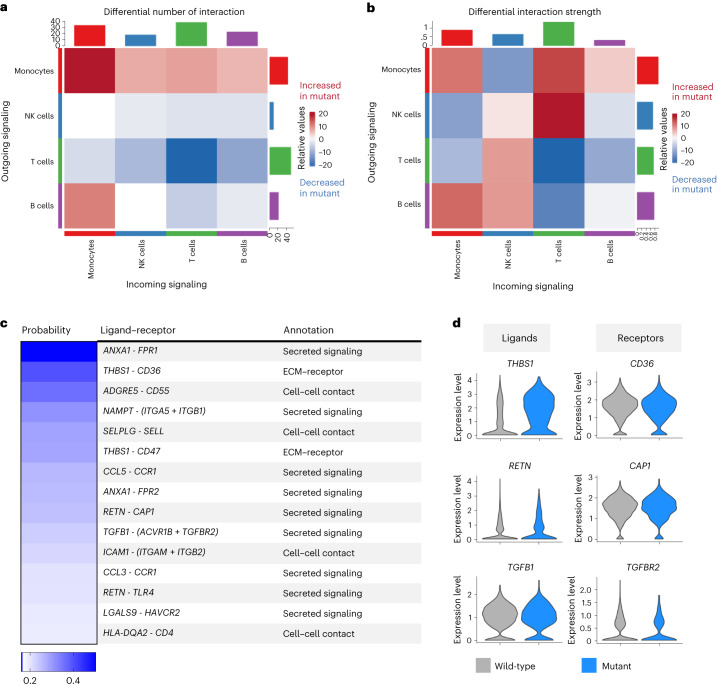
Interactions between immune cells with and without *DNMT3A* mutations. **a**,**b**, Heat map of outgoing versus incoming communication signal numbers (**a**) and strengths (**b**) among different cell types colored by relative interaction of mutant cells (red: increased interaction in mutant cells; blue: decreased interaction in mutant cells). **c**, Predicted monocyte–monocyte interactions sorted by highest interaction probability showing ligand–receptor pairs from mutant cells, type of signaling and evidence for the annotation. **d**, Relative expression of ligand and receptor in mutant and wild-type cells, respectively. ECM, extracellular matrix.

**Extended Data Fig. 8:**
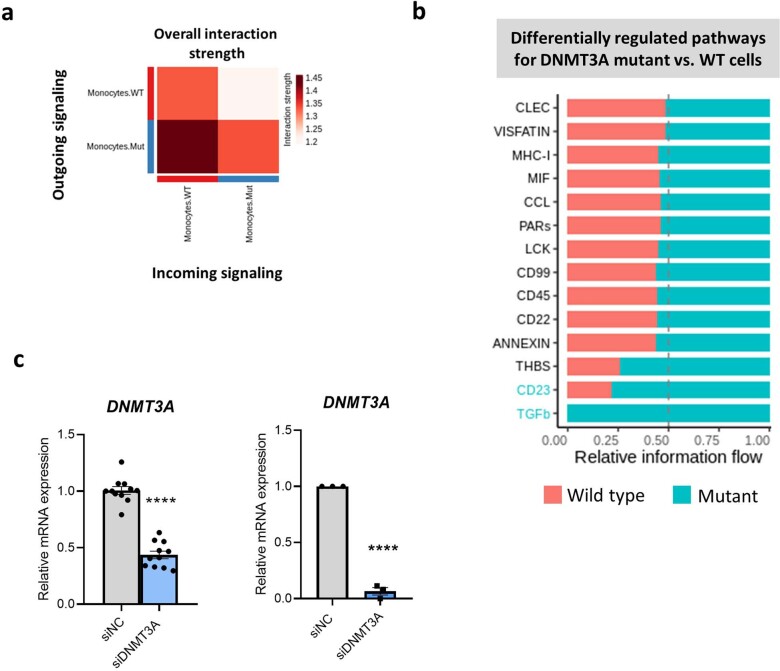
Changes in signaling by DNMT3A mutation presence. **a**. Interaction strength between wild type and *DNMT3A* mutant monocytes. Overall interaction strength is shown **b**. Differentially regulated pathways for *DNMT3A* mutant vs. WT cells. **c**. *DNMT3A* expresssion after *DNMT3A* silencing in human macrophages (n = 11 biologically independent samples, 4 donors), P = 1,63 × 10^−10^ (left) and in THP1-derived macrophages (n = 3 independent experiments), P = 9,59 × 10^−6^ (right). Means ± SEM is shown. Statistical significance was determined by two-tailed unpaired t-test. (**** *P* < 0.0001). Source data

Our data support a cell-intrinsic augmented inflammatory signature in *DNMT3A* mutant monocytes and T cells. However, the number of mutated cells in patients is low, and it is unclear how such small numbers of activated cells can impact the prognosis of heart failure^31^. This raises the question of whether mutant cells indirectly promote the inflammatory response by activating wild-type cells. To address this question, we analyzed the outgoing and incoming signaling strengths in mutant and wild-type cells by performing a ligand–receptor analysis with CellChatDB^32^ using all major annotated immune cell types. The differential interactions in the cell–cell communication networks between wild-type and mutant immune cells was determined and visualized, where red-colored edges represent increased signaling in the mutant immune cells compared to the wild-type immune cells, whereas blue color indicates decreased interactions (Fig. 5a,b). Thereby, both the numbers of interactions (Fig. 5a) and interaction strength (Fig. 5b and Extended Data Fig. 8a) were increased in mutant monocytes compared to wild-type monocytes, NK cells and T cells. Mutant T cells showed less outgoing signaling changes relative to wild-type T cells (Fig. 5a,b).

We also compared the information flow (that is, the overall communication probability) across the mutant and wild-type immune cells with secreted extracellular factors such as thrombospondin 1 (THBS1) and TGF-β being implicated in mutant signaling (Fig. 5c,d and Extended Data Fig. 8a,b). Interestingly, these associations were driven by mutant monocyte interactions, which showed increased signaling and relative expression of *THBS1*, *NAMPT*, *CCL5*, *RETN* and *TGF-β* (Fig. 5c,d). Examples for significantly associated ligand–receptor interaction pairs are THBS1–CD36 or THBS1–CD47, RETN–CAP1 or RETN–TLR4 and TGFB1–TGFBR2 (Fig. 5c,d). Many of these interactions have known regulatory functions in monocytes. THBS1 interactions are involved in cell adhesion and the phagocytosis of apoptotic cells^33^; NAMPT is involved in monocyte/macrophage differentiation, polarization and migration^34^; RETN mediates monocyte recruitment and promotes inflammation^35^; and TGF-β is well known for its pro-fibrotic activity^36^. These data suggest that mutant monocytes can interact and possibly activate wild-type monocytes in a paracrine manner.

**Fig. 6:**
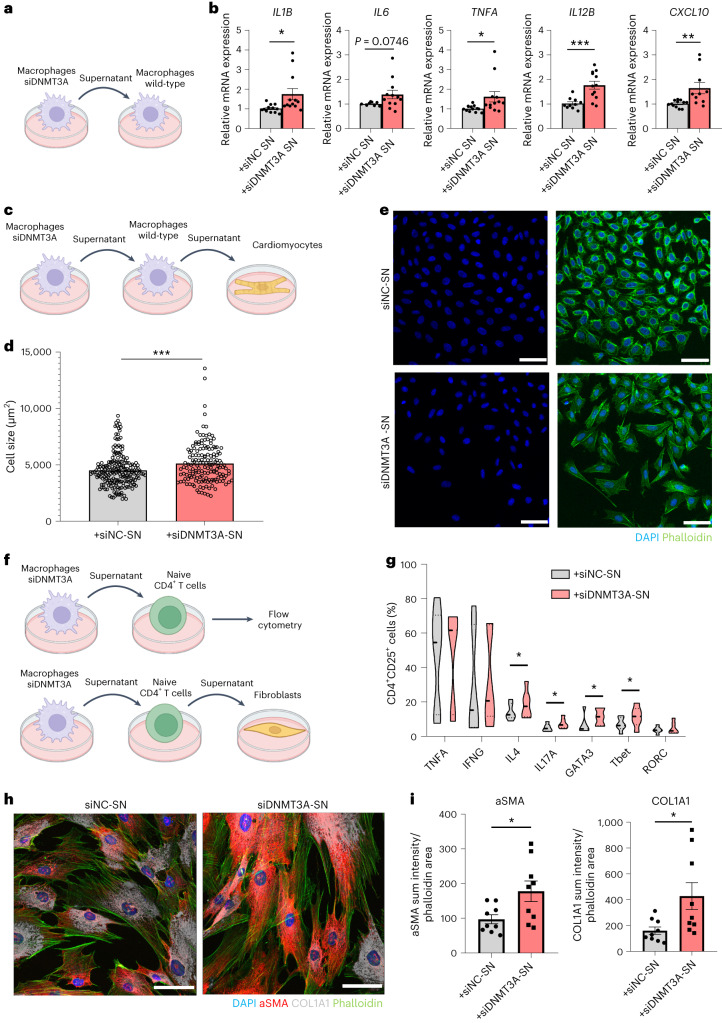
*DNMT3A*-silenced macrophages activate wild-type immune cells and cardiac cells by paracrine signaling. **a**, Experimental procedure to analyze capacity of *DNMT3A*-silenced macrophages to activate wild-type macrophages. **b**, Expression of pro-inflammatory markers in human primary macrophages after indirect co-culture with *DNMT3A*-silenced macrophages (*n* = 12 biologically independent samples except for *IL6* in siNC, *n* = 9, for *IL12B* in siNC and for *CXCL10* in siDNMT3A, *n* = 10, four donors), *P* = 0.0193, 0.0339, 0.0008 and 0.0077 for *IL1B*, *TNFA*, *IL12B* and *CXCL10*, respectively. **c**, Visualization of procedure to analyze indirect activation of cardiomyocytes by *DNMT3A*-silenced macrophages via wild-type macrophages. **d**, Immunofluorescence quantification of cardiomyocyte cell size after treatment with supernatant from THP1-derived macrophages indirectly co-cultured with *DNMT3A*-silenced macrophages (*n* = 203 and 156 biologically independent cells for siNC and siDNMT3A, four independent experiments), *P* = 0.0007. **e**, Representative immunofluorescence analysis of cardiomyocytes stained with DAPI (blue) and phalloidin (green) to quantify hypertrophic effects (scale bar, 50 µm). **f**, Experimental procedure to analyze capacity of *DNMT3A*-silenced macrophages to activate wild-type T cells (top) and cardiac fibroblasts (bottom). **g**, Flow cytometry analysis of naive CD4^+^ T cells after indirect co-culture with *DNMT3A*-silenced human macrophages (*n* = 6 donors), *P* = 0.0370, 0.0480, 0.0280 and 0.0433 for IL4, IL17, GATA3 and T-bet, respectively. **h**, Representative immunofluorescence analysis of cardiac fibroblasts after treatment with supernatant from CD4^+^ T cells indirectly co-cultured with *DNMT3A*-silenced human macrophages. DAPI (blue), phalloidin (green), collagen type I (gray) and αSMA (red) are stained (scale bar, 50 µm). **i**, Immunofluorescence quantification of COL1A1 and aSMA in cardiac fibroblasts (*n* = 9 biologically independent samples, three independent experiments), *P* = 0.0242 and 0.0255 for αSMA and COL1A1. Data show means ± s.e.m. (**b**,**d**,**i**) or medians (**g**). Statistical significance was assessed by two-tailed unpaired (**b**,**i**) or paired (**g**) *t*-test and two-tailed Mann–Whitney test (**d**) (*P < 0.05, **P < 0.01, ***P < 0.001). Source data

To determine the biological relevance of our in silico findings, we investigated whether *DNMT3A* silencing might indeed lead to a paracrine activation of wild-type monocyte-derived macrophages (Fig. 6a and Extended Data Fig. 8c). Therefore, we tested the effects of supernatants of *DNMT3A-*silenced macrophages on wild-type macrophages and showed that the supernatants indeed induced the expression of prototypical pro-inflammatory genes (for example, *IL1B*, *IL6* and *TNFA*) (Fig. 6b). To next assess if the macrophages, which are activated by the supernatants from *DNMT3A*-silenced macrophages, may contribute to cardiac dysfunction, we determined their paracrine effects on cardiomyocytes (Fig. 6c). Indeed, the supernatants of the pre-activated macrophages increased the cell size of cardiomyocytes (siNC supernatant-treated mean size: 4,512 µm^2^; siDNMT3A supernatant-treated mean size: 5,118 µm^2^) (Fig. 6d,e). These data demonstrate that *DNMT3A* mutant macrophages indirectly can promote pro-hypertrophic activity of wild-type macrophages.

**Fig. 7:**
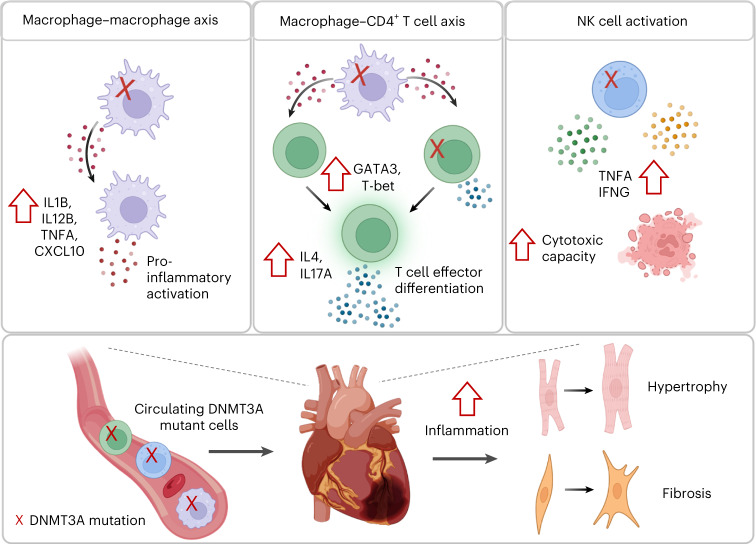
Crosstalk between immune cells with and without *DNMT3A* mutations amplifies inflammation contributing to cardiac dysfunction. Circulating blood cells are recruited to the heart after myocardial infarction. *DNMT3A* mutant macrophages promote pro-inflammatory activation of wild-type macrophages (macrophage–macrophage axis) and effector differentiation of CD4^+^ T cells (macrophage–CD4^+^ T cell axis). *DNMT3A* mutant NK cells produce elevated levels of pro-inflammatory cytokines and show increased cytotoxic capacity. Both intrinsic activation of immune cells by *DNMT3A* mutations and indirect activation of wild-type cells by *DNMT3A* mutant macrophages promote progression of inflammation causing hypertophy and fibrosis, finally resulting in cardiac dysfunction.

Because our in silico analysis additionally predicted an increased interaction of mutant monocytes with T cells (Fig. 5a), we also determined the influence of the supernatants of *DNMT3A*-silenced monocyte-derived macrophages on CD4^+^ T cells (Fig. 6f). Naive CD4^+^ T cells stimulated with the conditioned medium of *DNMT3A*-silenced macrophages expressed increased levels of cytokines (IL-4 and IL-17) and transcription factors (T-bet and GATA3) indicative of CD4^+^ T cell differentiation (Fig. 6g and Extended Data Fig. 6b). Because an increased polarization of T cells, particularly Th17 cells, is associated with cardiac fibrosis in humans^37,38^, we analyzed the capacity of CD4^+^ T cells exposed to supernatants from *DNMT3A*-silenced macrophages to activate human cardiac fibroblasts (HCFs) (Fig. 6f). Therefore, fibroblasts were cultured in the presence of supernatants from stimulated CD4^+^ T cells. Fibroblasts showed increased expression of the activation marker αSMA and COL1A1 after exposure to supernatants from CD4^+^ T cells stimulated with conditioned medium from *DNMT3A-*silenced macrophages (Fig. 6h,i). Together, these data suggest that *DNMT3A* mutant macrophages can activate wild-type macrophages and CD4^+^ T cells, indirectly promoting hypertrophy and fibrosis of cardiac cells in a paracrine manner (Fig. 7).

## Discussion

Here we provide novel insights into the cell-intrinsic effect of *DNMT3A* mutations on circulating blood cells in the context of heart failure. Our data demonstrate that *DNMT3A* mutant cells can be retrieved in all major cell populations, a finding that is consistent with previous reports documenting the multipotent pattern of *DNMT3A* CHIP mutations and lack of effects on blood cell counts^12^. Mutant cells were not enriched in any of the cell types and did not form a new entity but accumulated throughout the clusters, which is consistent with findings in CD34^+^cells^2^. However, *DNMT3A* mutations induced a substantially altered gene expression in the affected cells compared to wild-type cells in the same individual. It should be noted that, by comparing circulating cells from patients with established heart failure with versus without *DNMT3A* CHIP driver mutations, the observed effects are demonstrated on top of the presence of heart failure, which, by itself, is known to be associated with inflammatory activation of circulating blood cells. Dysregulated gene expression was predominantly observed in monocytes and NK cells, whereas only limited effects of *DNMT3A* mutants on T cells were observed. However, when subsetting the diverse T cell populations into CD4^+^ and CD8^+^ T cells, gene expression was considerably altered in the CD4^+^ T cell population. Here, *DNMT3A* mutant cells showed gene expression profiles that are indicative of T cell activation and increased effector functions. These findings were substantiated by showing that silencing of *DNMT3A* leads to the induction of Th1-, Th2- and Th17-related markers in CD4^+^ T cells, when additional cytokines were added to stimulate T cell polarization. It would be interesting to further gain insights into the gene expression profiles of the specific T cell populations in patients carrying *DNMT3A* mutations; however, this study does not have sufficient numbers of wild-type and mutant cell numbers to perform in-depth analyses on these divergent populations. The direct effects of *DNMT3A* on the activation of T cells observed in the in vitro studies may, however, be particularly relevant in cardiac disease, given that T cells, specifically Th17 cells, are known to aggravate heart failure^39,40^. Most notably, we also showed that *DNMT3A* mutations or *DNMT3A* silencing promoted NK cell effector functions and increased their cytolytic activity toward endothelial cells. NK cells are less well studied, but clinical abnormalities in the numbers and functions of NK cells are observed in myocarditis and inflammatory dilated cardiomyopathy, suggesting their role as regulators of cardiac function. Additionally, it has been shown that NK cells, via T-bet and IFNG signaling, play an essential role in angiotensin II-induced vascular dysfunction, driving increased NK cell recruitment and endothelial dysfunction^41,42^.

The differentially expressed genes in mutant monocytes confirmed previous studies showing a pro-inflammatory activation of myeloid cells after *DNMT3A* gene editing or depletion^7^. Also, many of the genes, such as S100 family members, were observed in the overall circulating cell population of patients with heart failure who carry a *DNMT3A* mutation versus NO-CHIP carriers^8^. Overlap in genes of total peripheral blood analysis of CHIP versus NO-CHIP patients with cell-intrinsic regulation of mutant cells suggests that the mutant cells are indeed the major driver in altered gene expression signatures found in CHIP patients despite representing only a few percent of the total cell population. Our study additionally suggests that the mutant cells can aggravate inflammation in the wild-type cells via a paracrine mechanism. Specifically, *DNMT3A-*silenced macrophages induced the pro-inflammatory activation of wild-type macrophages, which further induced hypertrophy of cardiomyocytes. Moreover, the supernatants of *DNMT3A-*silenced macrophages stimulated T cell polarization and stimulated the capacity of the T cells to activate fibroblasts. These paracrine activation mechanisms induced by the relatively small number of mutant cells may contribute to the progression of heart failure shown in clinical studies^4,5^. Interestingly, cell communication analysis identified thrombospondin, resistin, NAMPT and TGF-β as important drivers in cellular interaction. All these genes have known effects in monocytes and may further fuel inflammation. However, many have also bidirectional and context dependent effects^43^, and in vivo studies are warranted to determine the specific roles of these genes in mediating pathological effects aggravated by *DNMT3A* CHIP in the context of heart failure.

In addition to providing, to our knowledge, the first evidence for the cell-intrinsic effects of *DNMT3A* mutations on peripheral blood cell subsets in patients with heart failure, the experimental approach may be helpful to address the effects of the various CHIP driver genes in a disease context. Given that mutations of multiple genes appear to be linked to heart failure^44^, this may allow one to determine whether a common pro-inflammatory signature underlies the detrimental effects of the mutations or whether mutation-specific gene expression and functional effects can be observed. This also may have therapeutic consequences as it may lead to mutation-specific treatment strategies. Patient-specific and mutation-specific treatment is routinely done in the cancer field but is sparse in the treatment of cardiovascular disease. As such, this study may, in the long run, offer increased opportunities.

## Methods

The study adheres to all relevant ethical guidelines and was approved by the local ethics review board (Ethikkommission des Fachbereichs Medizin der Goethe-Universität) and complies with the Declaration of Helsinki.

### scRNA-seq study population and blood collection

Blood was obtained of patients with chronic heart failure at approximately the same time (between 10:00 and 12:00) on study days. Informed consent was obtained from all patients. The study was approved by the local ethics review board and complies with the Declaration of Helsinki. Patients were eligible for inclusion into the study if they had stable chronic heart failure symptoms New York Heart Association (NYHA) classification of at least II and had a previous myocardial infarction at least 3 months before recruitment. Exclusion criteria were the presence of acutely decompensated heart failure with NYHA class IV, an acute ischemic event within 3 months before inclusion, a history of severe chronic disease, documented cancer within the preceding 5 years or unwillingness to participate. Blood was collected in sodium citrate–containing cell preparation tubes (CPT Vacutainer, Becton Dickinson), which were centrifuged at 1,500*g* for 20 min at room temperature. The upper layer containing mononuclear cells and plasma was collected, and cells were washed and then stored until used for droplet scRNA-seq. The six patients in this study were male, averaged 66 years of age, gave consent for this study and were not compensated.

### scRNA-seq library preparation

Cellular suspensions were loaded on a 10x Chromium Controller (10x Genomics) according to manufacturer’s protocol based on the 10x Genomics proprietary technology. scRNA-seq libraries were prepared using Chromium Single Cell 3′ Reagent Kit version 3 (10x Genomics) according to the manufacturer’s protocol. In brief, the initial step consisted of performing an emulsion capture where individual cells were isolated into droplets together with gel beads coated with unique primers bearing 10x cell barcodes, unique molecular identifiers (UMIs) and poly(dT) sequences. Reverse transcription reactions were engaged to generate barcoded full-length cDNA followed by the disruption of emulsions using the recovery agent and cDNA cleanup with Dynabeads MyOne Silane Beads (Thermo Fisher Scientific). Bulk cDNA was amplified using a Biometra Thermocycler TProfessional Basic Gradient with 96-well sample block (98 °C for 3 min; cycled 14×: 98 °C for 15 s, 67 °C for 20 s and 72 °C for 1 min; 72 °C for 1 min; held at 4 °C). Amplified cDNA product was cleaned with the SPRIselect Reagent Kit (Beckman Coulter). Indexed sequencing libraries were constructed using the reagents from the Chromium Single Cell 3′ Reagent Kit version 3, as follows: fragmentation, end repair and A-tailing; size selection with SPRIselect; adaptor ligation; post-ligation cleanup with SPRIselect; sample index polymerase chain reaction (PCR) and cleanup with SPRIselect beads. Library quantification and quality assessment was performed using Bioanalyzer Agilent 2100 using a High Sensitivity DNA Chip (Agilent). Indexed libraries were equimolarly pooled and sequenced on two Illumina NovaSeq 6000 instruments using paired-end 26 × 98-bp as sequencing mode.

For preparing the scRNA-seq library of THP1 samples, the cells were labeled following Cell Multiplexing Oligo (CMO) Labeling for Single Cell RNA Sequencing Protocols with Feature Barcode technology (10x Genomics). In brief, 0.2 × 10^6^ cells of each sample were labeled with the CMOs. After the washing steps, the cells were additionally labeled using the TotalSeq-B Human Universal Cocktail version 1.0 (BioLegend), following the manufacturer’s instructions. The cellular suspension was loaded on a 10x Chromium Controller (10x Genomics) and processed following Chromium Next GEM Single Cell 3′ Reagent Kits version 3.1 (Dual Index) with Feature Barcode technology for Cell Surface Protein and Cell Multiplexing. cDNA was generated using an 11-cycle program (described above). Then, 3′ Gene Expression Library, Cell Surface Protein Library and Cell Multiplexing Library were generated using 13-, 10- and 6-cycle programs, respectively.

FASTQ files were processed using Cell Ranger 7.0.0 (10x Genomics) and aligned to the human reference genome GRCh38 according to the manufacturer’s instructions.

### CHIP gene capture probe design

A target enrichment library (Roche–NimbleGen) was designed by obtaining gene annotations of CHIP-associated genes *DNMT3A* and *TET2*. For each gene, genome coordinates of their corresponding exons were obtained from the GRCh38 primary assembly. Design of probes from target regions and synthesis was performed by Roche–NimbleGen using the SeqCap RNA Choice format with a maximum of five matches to the human genome. In total, 34 exons were targeted by the CaptureSeq array.

### Single-cell targeted enrichment

Before targeted enrichment, a fraction of amplified full-length cDNA from 10x Genomics library construction (2–5 ng) was re-amplified with KAPA HiFi HotStart ReadyMix (Roche) and 3 µM of each TSO primer NNNAAGCAGTGGTATCAACGCAGAGTACAT and R1 primer NNNCTACACGACGCTCTTCCGATCT. Adopted from previous work^10^, the Ns at the primer 5′ end were introduced to avoid preferential amplification of reverse reads. The cycling conditions were as follows: initial denaturation at 98 °C for 3 min, followed by 20 cycles of 98 °C for 30 s, 64 °C for 30 s and 72 °C for 5 min and a final elongation at 72 °C for 10 min. PCR products were purified with 0.6× SPRIselect (Beckmann Coulter), eluted with 40 µl of nuclease-free water and quantified with a Qubit 4 Fluorometer (Thermo Fisher Scientific). To enrich transcripts of interest, the amplified full-length cDNA (0.5–1 µg) was subjected to two sequential hybridization and capture reactions following the KAPA HyperCap Workflow version 3.0 (Roche). In brief, the cDNA library was hybridized to 2 µl of biotinylated KAPA HyperChoice Costum Probes (Roche) at 55 °C for 16 h, excluding Universal Enhancing Oligos, and incubated with Capture Beads for 15 min. A series of washes was performed to remove unbound cDNA. After the first capture, the enriched cDNA library was amplified with KAPA HiFi HotStart ReadyMix (Roche) and 1 µM of each TSO and R1 primer in an on-bead PCR for five cyles (98 °C for 3 min, cycles of 98 °C for 30 s, 64 °C for 30 s and 72 °C for 5 min and 72 °C for 10 min). The enriched cDNA library underwent a second hybridization and capture at similar conditions, followed by an on-bead PCR for 25 cycles with two amplification reactions per sample. After each post-capture amplification, the Capture Beads were magnetically removed from the PCR reaction, and the enriched cDNA library was cleaned up with KAPA HyperPure Beads. Finally, the enriched cDNA library was analyzed on a High Sensitivity DNA Chip (Agilent), and the enrichment of targeted transcripts was assessed by quantitative real-time PCR (qPCR) before Oxford Nanopore library preparation.

### Oxford Nanopore long-read sequencing

Nanopore sequencing libraries were prepared from 200 fmol targeted capture cDNA library using the Ligation Sequencing Kit (SQK-LSK110) and the Amplicons by Ligation Protocol (ONT). Adapter-ligated libraries (20–50 fmol) were loaded on MinION106D (R9.4.1) flow cells and sequenced on either an Oxford Nanopore Mk1C device with MinKNOW Software at Goethe University or an Oxford Nanopore PromethION device by FutureGenomics. Base calling was performed offline on a high-performance computing cluster using ONT’s Albacore software pipeline (version 2.2.7).

### Nanopore read mapping

Reads from the Nanopore sequencing were mapped to the human reference genome (GRCH38) via minimap2 version 2.24 in splice alignment mode with the following parameters: ‘-ax splice -uf–MD–sam-hit-only -t 20–junc-bed’. Before the cellBc and UMIs were assigned, we added the gene name, nanopore read sequence and the read quality to the SAM record via the Sicelore companion tools AddGeneNameTag and AddBamReadSequenceTag, as proposed in the Sicelore protocol^10^.

### cellBC and UMI assignment to Nanopore reads

To assign cellBC and UMI sequences to the mapped Nanopore reads, we basically followed the Sicelore protocol suggested by Lebrigand et al.^10^. In brief, valid cellBC and UMIs were extracted from the single-cell BAM file with the IlluminaParser tool. Afterwards, Nanopore reads are parsed for poly-(A/T) and adapter sequences via NanoporeReadScanner. Only reads containing both were kept for further processing. After the Nanopore reads were mapped as described in the previous subsection, cellBC and UMIs were searched in the BAM file, with an allowed edit distance of 1. If successful, the tool NanoporeBC_UMI_finder adds both sequences as flags to the SAM/BAM record. To reduce the impact of sequencing errors, reads with identical mapping positions in the target gene and identical UMI barcodes were used to generate a consensus sequence. For this purpose, the ComputeConsensus tool was used, with default parameters. These consensus sequences are then mapped to the human reference genome. The previously detected single-nucleotide polymorphisms (SNPs) were then individually searched in the patients using the tool SNPMatrix. A cell was assigned as ‘mutated’ if only the mutated allele could be detected or the mutated allele was detected along with the original allele. If no sequences were detected at the mutation site, the reads were assigned as ‘no-coverage’. If only the native alleles could be found, the cells were assigned as ‘non-mutated’. Finally, the mutation information for each patient was assigned the cells in the Seurat objects with the function combineSeuratandSicelore. All steps described in the above subsection were automatized in the run_sicelore.sh bash script. All scripts and source codes can be found at https://github.com/djhn75/Sicelore-scRNA-Integration.

### Short-read scRNA-seq

Each library was sequenced at high coverage on a NovaSeq 6000 sequencing system (Illumina) using the paired-end 150-bp approach (Illumina) as specified by the manufacturer. BCL files were converted and demultiplexed in BaseSpace by Illumina.

### scRNA-seq data analyses

Single-cell expression data were processed using STARsolo (version 2.7.3a) to perform quality control, barcode processing and single-cell 3′ gene counting. Sequencing reads were aligned to the human reference genome (GRCh38). STARsolo was run with default parameters, and quality and content of the sequenced libraries were assessed (Extended Data Table 2). Further analysis was performed in Seurat (version 4) R (version 3.6). Data were filtered based on the number of genes detected per cell (cells with fewer than 200 genes per cell were filtered). A global-scaling normalization method for the gene–cell–barcode matrix of the samples was employed. We normalized the feature expression measurements for each cell by the total expression, multiplied this by a scale factor (10,000) and log-transformed the result to yield the normalized unique molecular identifier (nUMI) value later reported. Regression in gene expression was performed based on the number of nUMIs. Principal component analysis (PCA) was run on the normalized gene–barcode matrix. Barnes–Hut approximation to UMAP was then performed on principal components to visualize cells in a two-dimensional space. This graph-based clustering method relies on a clustering algorithm based on shared nearest neighbor (SNN) modularity optimization. Differential transcriptional profiles by cluster were generated in Seurat with associated GO terms derived from the functional annotation tools DAVID Bioinformatics Resources 6.7 (NIAID/NIH, https://david.ncifcrf.gov/summary.jsp) and Metascape (http://metascape.org). For THP-1 analyses, first *DNMT3A* knockdown efficiency was assessed to detect siRNA responsive and non-responsive cells. Within the siRNA treatment, there were three ranges of data based on *DNMT3A* expression (no expression, low expression and high expression). As 10x Genomics data have gene dropout issues, we considered for analysis only cells with detectable *DNMT3A* and stratified these by high and low *DNMT3A* expression, which is termed responsive and non-responsive. Ligand–receptor interaction was performed per standard pipelines using the CellChatDB tool. SCENIC was used to assess transcription factor regulation implicated in *DNMT3A-*mutated cells. Cell annotation was then performed by assessing relative expression of immune markers as described in the Results section. To perform differential abundance testing between mutated and unmutated cells for each cell type, we applied MILO^45^ according to their vignettes. In brief, the Seurat object was transformed to a SingleCellObject by as.SingleCellExperiment(), followed by a PCA with RunPCA() and an UMAP reduction via RunUMAP(). The resulting object was then imported to MILO, and a *k*-nearest neighbor (KNN) graph was generated via buildGraph() with the parameters *k* = 10 and *d* = 30. Afterwards, representative neighborhoods were defined with makeNhoods() with the parameters *prop* = 0.1, *k* = 10 and *d* = 30. Based on the cell type and mutation information, the cells for each KNN neigborhood were counted by countCells(). Finally, differential abundance testing was performed by calcNhoodDistance() and testNhood().

### Variant calling and annotation strategies for targeted DNA sequencing

Read quality was assessed using FastQC. FASTQ files from each patient were merged, and reads were grouped into UMI families using the UMI-tools software package (version 1.1.2). Reads were mapped to the hg19 draft of the human genome using Burrows–Wheeler Alignment–MEM. The ‘dedup‘ command of the UMI-tools software package was used to remove PCR duplicates with the same UMIs and alignment coordinates. Variant calling was performed using FreeBayes without allele frequency threshold, a minimum alternate read count of 2 and a minimum base and mapping quality of 20. Variant effect prediction and variant annotation were performed using SnpEff and SnpSift. The identified variants were processed and filtered using the R programming language, version 3.3.1. Common SNPs with a minor allele frequency of at least 5% in the 1000 Genomes Project, Exome Variant Server or ExAC databases were excluded. In addition, variants with allow mapping quality (<20) and variants occurring in 8% or more of the patients in the studied cohort were considered as technical artifacts and excluded. Furthermore, variants covered with fewer than 100 reads in at least one set of amplicons (CAT A or CAT B), variants called with only one of the set of amplicons (CAT A or CAT B), SNPs identified as common in the SNP database (≥1% in the human population) and variants with sequence ontology terms ‘LOW’ or ‘MODIFIER’ were filtered out. According to previously established definitions, all variants with a VAF of at least 0.01 (1%) were considered; VAF was calculated by using the formula VAF = alternate reads / (reference + alternate reads). Variants with a VAF of 0.45–0.55 were not considered, to exclude potential germline variants. The variants were further validated on the basis of being reported in the literature and/or the Catalogue of Somatic Mutations in Cancer (https://cancer.sanger.ac.uk/cosmic) and ClinVar (https://www.ncbi.nlm.nih.gov/clinvar). Four patients had a mutation in the MTPase region; one patient had a deletion in the MTPase region; and one patient exhibited a frame shift (amino acid 449).

### Cell culture and siRNA transfection

THP1 cells were purchased from the German Collection of Microorganisms and Cell Cultures (ACC16), and the NK cell line NKL was a kind gift from Winfried Wells (Georg-Speyer-Haus). Human cardiomyocyte ventricular (HCM-VT) primary cells were obtained from Celprogen (36044-15VT), and primary HCFs (C-12375) were from PromoCell. HUVECs were purchased from Lonza (C2519A). Human primary immune cells were isolated from PBMCs derived from buffy coats of healthy donors.

Immune cell lines and primary cells were cultured in RPMI 1640 media (11875093, Thermo Fisher Scientific) with 10% heat-inactivated FBS (10082147, Thermo Fisher Scientific), 1% penicillin–streptomycin (15140122, Thermo Fisher Scientific), 1% glutamin (35050061, Thermo Fisher Scientific) (basic RPMI) and cell-type-specific additives at 37 °C and 5% CO_2_. THP1 cells, NKL cells and primary cells isolated from peripheral blood were cultured in basic RPMI supplemented with 10 mM HEPES (H0887, Sigma-Aldrich), with 200 U ml^−1^ IL-2 (200-02, PeproTech) and with 1% sodium pyruvate (S8636, Sigma-Aldrich) and 50 µM β-mercaptoethanol (M3148, Sigma-Aldrich), respectively. HCM-VT cells were kept in Human Cardiomyocyte Complete Media with Serum (M36044-15S, PromoCell), and HCFs were maintained in Fibroblast Growth Medium 3 (C-23130, PromoCell) according to the manufacturer’s protocol. HUVECs were cultured in endothelial basal medium (CC31221) supplemented with EGM BulletKit components (CC-3124) containing hydrocortisone (1 μg ml^−1^), bovine brain extract (12 μg ml^−1^), gentamicin (50 μg ml^−1^), amphotericin B (50 ng ml^−1^), epidermal growth factor (10 ng ml^−1^) and FBS (2%).

Silencing of target genes was performed with siRNA and siTrans2.0 transfection reagent (T320001, OriGene) according to the manufacturer’s protocol. *DNMT3A* was silenced using stealth siRNA (1299001, HSS176225) and a corresponding negative control of median GC content (12935300, Invitrogen).

### Preparation of PBMCs

PBMCs were isolated from buffy coats of healthy donors, obtained from Blutspendedienst Frankfurt, by density gradient centrifugation. In brief, PBMC-enriched blood was diluted 1:3 with pre-warmed PBS supplemented with 0.5% BSA (A1595, Sigma-Aldrich) and 2 mM EDTA (E8008, Sigma-Aldrich) and layered on top of human Pancoll (1.077 g ml^−1^, P04-60500, PAN Biotech), followed by centrifugation at 400*g* for 25 min with disabled break. The PBMC layer was collected, washed and centrifuged at 300*g* for 10 min. Platelets were removed by two additional washing steps and centrifugation at 200*g* for 10 min. Cell types of interest were enriched by magnetic-activated cell sorting (MACS, Miltenyi Biotec). Monocytes and naive CD4^+^ T cells were isolated using the human Pan Monocyte Isolation Kit (130-096-537) and the Naive CD4^+^ T Cell Isolation Kit II (130-094-131), respectively (all from Miltenyi Biotec), following the manufacturer’s guidelines. The enrichment of desired cell types was confirmed by flow cytometry.

### Monocyte-derived macrophages

Monocytes freshly isolated from PBMCs were seeded in six-well plates (1.5 × 10^6^ cells per well) and allowed to adhere in non-supplemented RPMI 1640 media for 2 h. Macrophage differentiation was induced in complete media with 20 ng ml^−1^ GM-CSF (300-03, PeproTech), culturing cells in the presence of GM-CSF for 6 d with a media exchange after 3 d. After the differentiation was completed, macrophages were transfected with 100 nM siRNA, refreshing the media after 6 h. THP1 monocytes were differentiated into THP1-derived macrophages with 100 ng ml^−1^ phorbol 12-myristate 13-acetate (PMA) (P8139, Sigma-Aldrich) for 48 h, followed by 24 h of culture in media without PMA. THP1 macrophages were transfected with 50 nM siRNA, and the media were refreshed 16 h after transfection.

For indirect co-culture experiments, the supernatant of siRNA-transfected macrophages was collected after 48 h, diluted 1:2 and applied on primary wild-type macrophages for 24 h. Wild-type THP1 macrophages were indirectly co-cultured with siRNA-transfected THP1 macrophages for 72 h. The supernatants of stimulated wild-type macrophages were diluted 1:2 with cardiomyocyte media and applied on HCM-VT cells for 48 h.

### Differentiation of naive CD4^+^ T helper cells

After MACS isolation, naive CD4^+^ T cells were seeded in 48-well plates (2 × 10^5^ cells per well) and rested for 3 h before transfection with 100 nM siRNA. The media were refreshed 16 h after transfection, followed by T cell activation and differentiation into T helper cell subsets. Naive T cells were activated in the presence of 5 ng ml^−1^ IL-2 (200-02, PeproTech) and CD3 and CD28 agonists (T Cell TransAct, 130-111-160, Miltenyi Biotec) to generate Th0 cells. Th1, Th2 and Th17 cells were induced with 20 ng ml^−1^ IL-12 (200-12), 20 ng ml^−1^ IL-4 (200-04) and 5 ng ml^−1^ TGF-β (100-21C), plus 50 ng ml^−1^ IL-6 (200-06), respectively (all cytokines from PeproTech). In addition, polarizing cytokines were replaced by supernatant from siRNA-transfected human primary macrophages used in 1:5 dilution for CD4^+^ T cell differentiation. Cells were differentiated for 6 d with a media exchange after 3 d. Supernatants of CD4^+^ T cells indirectly co-cultured with human macrophages were collected from day 3 to day 6 of differentiation, diluted 1:2 with HCF media and used to stimulate HCFs for 48 h.

### RNA extraction and qPCR

For qPCR analysis, intron-spanning primers were designed using the Primer BLAST tool. Primers were confirmed to amplify a specific product meeting qPCR efficiency of 100 ± 15%. All qPCR primers are listed in Supplementary Table 1. SYBR Green-based assays were performed in a 10-µl reaction consisting of 5 µl of Fast SYBR Green Master Mix 2× (Applied Biosystems, 4385617, Thermo Fisher Scientific), 2 µl of nuclease-free water, 0.25 µl of each 10 mM forward and reverse primer and 10 ng of cDNA template in 2.5 µl. Assays were carried out in 384-well plates in technical triplicates and run on a ViiA 7 instrument with Quant Studio Real-Time PCR Software (both Applied Biosystems). The thermal profile consisted of 95 °C for 20 s, followed by 40 cycles of 95 °C for 1 s and 60 °C for 20 s and a final melt curve stage (95 °C for 15 s, 60 °C for 1 min and 95 °C for 15 s). Data were analyzed using the ΔΔCt method and RPLP0 as reference gene. Data are represented as relative mRNA level (2^−ΔΔCt^) normalized to the expression level in the respective control.

### Immunofluorescence

HCFs were seeded at a density of 5,000 cells per cm^2^ in eight-well µ-Slides (80826, ibidi) coated with human fibronectin (F0895, Sigma-Aldrich), and HCM-VT cells were seeded at a density of 200 cells per cm^2^ in eight-well µ-Slides for immunofluorescence stainings. After the respective treatment, cells were washed two times with DBPS and fixed with 4% paraformaldehyde (PFA) (28908, Thermo Fisher Scientific) for 10 min at room temperature. After two washing steps, each for 5 min, with DPBS, cells were permeabilized with 0.1% Triton X-100 (T8787, Thermo Fisher Scientific) in DPBS for 15 min. Cells were blocked with 5% donkey serum (ab7475, Abcam) in DPBS for 60 min at room temperature. Primary antibodies were incubated in the same blocking solution overnight at 4 °C. Cells were stained with phalloidin (1:100, O7466, Thermo Fisher Scientific), mouse anti-α-smooth muscle actin-Cy3 (1:200, C6198, Sigma-Aldrich) and rabbit anti-collagen type I (1:200, 72026, Cell Signaling Technology). Cells were washed four times with DPBS, each 5 min, and secondary antibodies in DPBS were incubated for 1 h at room temperature. The following dyes and secondary antibodies were used: DAPI (1:1,000, D9542, Merck) and donkey anti-rabbit-647 (1:200, A31573, Thermo Fisher Scientific). Cells were mounted with Fluoromount-G (00-4958-0, Invitrogen). Images were taken using a Leica STELLARIS confocal microscope and analyzed with Leica Application Suite X (2.0.1.14392). Distance between *z* acquisitions was defined to the half of the optical section of the objective used. Cardiomyocyte area was detected by the immunofluorescence signal for phalloidin and determined as the sum of areas acquired within the *z*-stack for each cell. Quantification was done using Volocity software version 6.5 (Quorum Technologies).

### Flow cytometry

Multicolor flow cytometry was performed to analyze surface markers in combination with intracellular transcription factors and secreted cytokines. The panels consisting of fluorochrome-conjugated antibodies in Supplementary Table 2 were applied. Potential spectral overlap in the emission of fluorochromes was compensated in advance with bulk PBMCs using unstained, single-stained, fluorescence-minus-one and complete stained controls.

For intracellular detection of secreted cytokines, differentiated CD4^+^ T cells were re-stimulated with 50 ng ml^−1^ PMA (P8139, Sigma-Aldrich) and 1 µg ml^−1^ ionomycin (I0634, Sigma-Aldrich) in the presence of 5 µg ml^−1^ Brefeldin A (420601, BioLegend) for 4 h. NK cells were treated with Brefeldin A for 4 h before intracellular cytokine staining. Then, cells were processed for flow cytometry analysis as follows. After washing with 2 ml of staining buffer (420201, BioLegend) and centrifugation at 400*g* for 5 min, cell surface antigens were stained in 100 µl of staining buffer. Afterwards, cells were washed with 2 ml of staining buffer, centrifuged at 400*g* for 5 min and fixed with an equal volume of IC Fixation Buffer (eBioscience, 00-8222-49, Thermo Fisher Scientific) for 20 min. After washing the cells with 2 ml of permeabilization buffer (eBioscience, 00-8333-56, Thermo Fisher Scientific), intracellular targets were stained in 100 µl of permeabilization buffer for 30 min. A final washing step with 2 ml of staining buffer was carried out before flow cytometry analysis. Data were acquired with a LSR Fortessa X-20 Cell Analyzer and FACSDiva Software (BD Biosciences); FCS files were analyzed with FlowJo Software (version 10.8.1).

### CFSE staining

To distinguish HUVECs from co-cultured NK cells, HUVECs were fluorescently labeled with CFSE (CellTrace, C34570, Thermo Fisher Scientific) according to the manufacturer’s instructions. In brief, after staining 1 × 10^6^ cells with 5 µM CFSE in 1 ml of PBS for 20 min at 37 °C, remaining dye in the solution was quenched with five volumes of complete media for 5 min. Then, the cells were pelleted and resuspended in pre-warmed complete media. Cells were analyzed by flow cytometry after staining after a resting period of at least 10 min. Before co-culture with NK cells, HUVECs were rested overnight.

### NK cell cytotoxicity assay

To study the cytotoxic capacity of NK cells on HUVECs, the NK cells were co-cultured with CFSE-labeled HUVEC target cells in a ratio of 1:1 for 4 h. Then, cells were trypsinized, washed with staining buffer and stained with 5 µl of 7-AAD (420404, BioLegend) for 15 min to identify dead cells. 7-AAD^+^ HUVEC cells were determined by flow cytometry. The percentage of lysed HUVECs was calculated as follows: %CFSE + 7-AAD + HUVEC (co-culture) − %CFSE + 7-AAD + HUVEC (HUVEC only).

### Reporting summary

Further information on research design is available in the Nature Portfolio Reporting Summary linked to this article.

### Supplementary information

Supplementary Tables 1 and 2

Reporting Summary

### Source data

Source Data Fig. 4 Statistical Source Data

Source Data Fig. 6 Statistical Source Data

Source Data Extended Data Fig./Table 8 Statistical Source Data

## Figures and Tables

**Extended Data Table 1:**
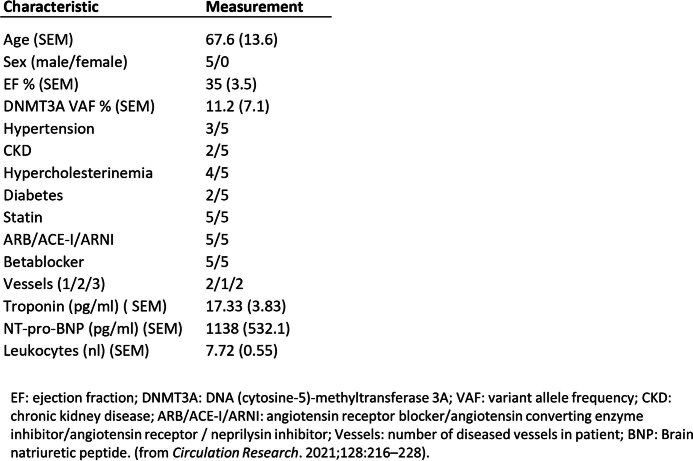
Patient characteristics

ARB/ACE-I/ARNI, angiotensin receptor blocker/angiotensin-converting enzyme inhibitor/angiotensin receptor-neprilysin inhibitor; BNP, brain natriuretic peptide; CKD, chronic kidney disease; EF, ejection fraction; Vessels, number of diseased vessels in patient (from *Circ. Res*. **128**, 216–228 (2021)).

**Extended Data Table 2:**
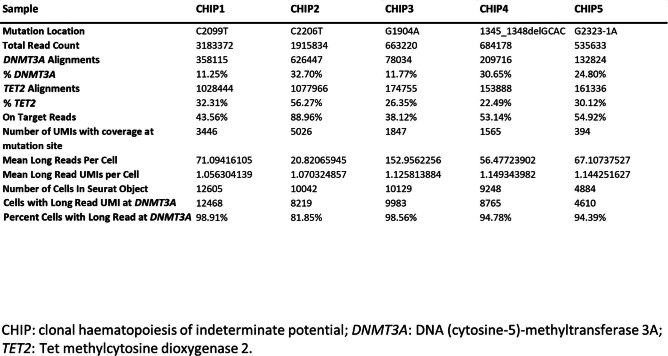
Long-read sequencing metrics of reads with detected cell barcodes from patient cohort

## Data Availability

Sequencing data supporting the findings of this study have been deposited in the ArrayExpress Data Depository with series accession number E-MTAB-13016. Data supporting the findings of this study are included in the main article and associated files and/or at the referenced depositories.

## Code availability

Scripts to reproduce the analysis presented in this study have been deposited on GitHub (https://github.com/djhn75/Sicelore-scRNA-Integration).
