# Supplementary material for: Cell-intrinsic effects of clonal hematopoiesis in heart failure
Source: Nat Cardiovasc Res. 2023 Sep 4;2(9):819–34. doi: 10.1038/s44161-023-00322-x (PMC11357996; doi:10.1038/s44161-023-00322-x)
Supplement: Supplementary file 1 — Supplementary Tables 1 and 2 [file 44161_2023_322_MOESM1_ESM.pdf]

nature cardiovascular research

Article https://doi.org/10.1038/s44161-023-00322-x

Cell-intrinsic effects of clonal hematopoiesis in heart failure

In the format provided by the authors and unedited

Supplementary Table 1. Oligonucleotides used in this study.

Primer	Sequence 5→3
R1-For	NNNCTACACGACGCTCTTCCGATCT
TSO-Rev	NNNAAGCAGTGGTATCAACGCAGAGTACAT
DNMT3a-For	TATTGATGAGCGCACAAGAGAGC
DNMT3a-Rev	GGGTGTTCCAGGGTAACATTGAG
TET2-For	AAGCAAGATCCCAAGGAAGT
TET2-Rev	GCAAATGAGACTCCAGTTT
CD3E-For	TGCCTCTTATCAGTTGGCGT
CD3E-Rev	TCCAGGATACTGAGGGCATGT
TRAC-For	GTGCAAACGCCTTCAACAAC
TRAC-Rev	CTCGACCAGCTTGACATCACA
TNFA-For	CATCCAACCTTCCCAAACGC
TNFA-Rev	CTGTAGGCCCCAGTGAGTTC
RPLP0-For	TCGACAATGGCAGCATCTAC
RPLP0-Rev	ATCCGTCTCCACAGACAAGG
IL1B-For	TCCAGCTACGAATCTCCGAC
IL1B-Rev	AGATGAAGGGAAAGAAGGTGC
IL6-For	AGACAGCCACTCACCTCTTC
IL6-Rev	TTTCACCAGGCAAGTCTCCT
IL12-For	AGCAGGTGAAACGTCCAGAA
IL12-Rev	AGCAGGTGAAACGTCCAGAA
CXCL10-For	GCTTCCAAGGATGGACCACA
CXCL10-Rev	GCAGGGTCAGAACATCCACT

Supplementary Table 2. Flow cytometry antibody panels.

Antibody	Fluorophore	Clone	Dilution	Cat	Company
**Naïve CD4+ T cell detection**					
CD4	BV786	OKT4	1:20	317442	BioLegend
CD45RA	AF700	HI100	1:20	304120	BioLegend
CD197 (CCR7)	BV605	G043H7	1:20	353224	BioLegend
**CD4+ T cells**					
CD4	FITC	RPA-T4	1:20	300506	BioLegend
CD3	AF700	OKT3	1:20	317340	BioLegend
CD25	PE-Cy5	BC96	1:20	302608	BioLegend
CD69	BUV395	FN50	1:20	564364	BD
TNFA	APC Cy7	Mab11	1:20	502944	BioLegend
IFNG	BV605	4SB3	1:20	502536	BioLegend
IL4	PE	MP4-25D2	1:100	500810	BioLegend
IL17A	BV786	N49-653	1:20	563745	BD
Tbet	BV421	4B10	1:20	644832	BioLegend
GATA3	BV711	L50-823	1:20	565449	BD
RORC	PE-CF594	Q21-559	1:20	567532	BD
**NK cells**					
CD56	FITC	MEM-188	1:20	304604	BioLegend
CD3	BV786	OKT3	1:20	317330	BioLegend
TNFA	APC Cy7	Mab11	1:20	502944	BioLegend
IFNG	BV711	4SB3	1:20	502540	BioLegend
