# Supplementary material for: Cell-intrinsic effects of clonal hematopoiesis in heart failure
Source: Nat Cardiovasc Res. 2023 Sep 4;2(9):819–34. doi: 10.1038/s44161-023-00322-x (PMC11357996; doi:10.1038/s44161-023-00322-x)
Supplement: Supplementary file 2 — Reporting Summary [file 44161_2023_322_MOESM2_ESM.pdf]

nature portfolio

Corresponding author(s): Stefanie Dimmeler

Last updated by author(s): Jun 22, 2023

Reporting Summary

Nature Portfolio wishes to improve the reproducibility of the work that we publish. This form provides structure for consistency and transparency in reporting. For further information on Nature Portfolio policies, see our Editorial Policies and the Editorial Policy Checklist.

Statistics

For all statistical analyses, confirm that the following items are present in the figure legend, table legend, main text, or Methods section.

n/a	Confirmed	
☐	☒	The exact sample size (*n*) for each experimental group/condition, given as a discrete number and unit of measurement
☐	☒	A statement on whether measurements were taken from distinct samples or whether the same sample was measured repeatedly
☐	☒	The statistical test(s) used AND whether they are one- or two-sided<br>*Only common tests should be described solely by name; describe more complex techniques in the Methods section.*
☒	☐	A description of all covariates tested
☐	☒	A description of any assumptions or corrections, such as tests of normality and adjustment for multiple comparisons
☐	☒	A full description of the statistical parameters including central tendency (e.g. means) or other basic estimates (e.g. regression coefficient) AND variation (e.g. standard deviation) or associated estimates of uncertainty (e.g. confidence intervals)
☐	☒	For null hypothesis testing, the test statistic (e.g. *F*, *t*, *r*) with confidence intervals, effect sizes, degrees of freedom and *P* value noted<br>*Give P values as exact values whenever suitable.*
☒	☐	For Bayesian analysis, information on the choice of priors and Markov chain Monte Carlo settings
☒	☐	For hierarchical and complex designs, identification of the appropriate level for tests and full reporting of outcomes
☒	☐	Estimates of effect sizes (e.g. Cohen's *d*, Pearson's *r*), indicating how they were calculated

Our web collection on statistics for biologists contains articles on many of the points above.

Software and code

Policy information about availability of computer code

Data collection	Data used are described in the supplementary method section. We used custom code which will be provided upon request and made public available after acceptance of the manuscript. Volocity 6.5, Leica Application Suite X 2.0.1.14392, FACS Diva 6.0.
Data analysis	Packages and software used are described in the supplementary method section. We used custom code found at https://github.com/djhn75/Sicelore-scRNA-Integration. Volocity 6.5, Graphpad 8, Seurat 4, FlowJo (Version 10.8.1, FlowJo LLC), QuantStudio Software (version 1.6, Thermo Fisher Scientific), Metascape 3.5, Cell Ranger Single Cell Software Suite 7.0.0, STARsolo (v 2.7.3a), UMI-tools software package (v.1.1.2)

For manuscripts utilizing custom algorithms or software that are central to the research but not yet described in published literature, software must be made available to editors and reviewers. We strongly encourage code deposition in a community repository (e.g. GitHub). See the Nature Portfolio guidelines for submitting code & software for further information.

Data

Policy information about availability of data

All manuscripts must include a data availability statement. This statement should provide the following information, where applicable:

- Accession codes, unique identifiers, or web links for publicly available datasets
- A description of any restrictions on data availability
- For clinical datasets or third party data, please ensure that the statement adheres to our policy

The single-cell RNA–seq data generated in this study are available at Array Express (https://www.ebHuman Reference Genome (GRCH38)i.ac.uk/arrayexpress) with the following accession numbers: E-MTAB-13016. All other data are included within the article, source data (for analyses with statistical tests reported), and supplementary data can be made available upon request. Source data are provided with this paper. The Human Reference Genome GRCH38 was used for alignment of long read sequencing data and scRNA-seq data while HG19 was first used for targeted DNA sequencing alignment of CHIP genes as previously reported (doi.org/10.1161/CIRCRESAHA.120.317104 and doi.org/10.1001/jamacardio.2018.3965).

Research involving human participants, their data, or biological material

Policy information about studies with human participants or human data. See also policy information about sex, gender (identity/presentation), and sexual orientation and race, ethnicity and racism.

Reporting on sex and gender	Most analyses were done with n=5 DNMT3A. Additional analysis was performed with n=1 TET2 (only Extended Data Figure 2) and n=4 No CHIP (only Extended Data Fig.4f) Research involving human research participants was performed in accordance with the Declaration of Helsinki.
Reporting on race, ethnicity, or other socially relevant groupings	Sociopolitical constructs such as race and ethnicity are not reported on in this manuscript.
Population characteristics	Age and sex are directly reported on. Previously published reporting characteristics are from Circulation Research. 2021;128:216–228. Average age of HF patients was 66 years old, all patients were male, and average ejection fraction of 36%. All patients were screened for presence of clonal hematopoiesis mutations and were required to have a DNMT3A somatic mutation in circulating immune cells. For the study patients, three patients had hypertension, two patients diagnosed with chronic kidney disease, five patients had hypercholesterinemia, two patients had diabetes, all patients were on statins and betablockers. C reactive protein ranged from 0.13 to 0.28 µg/mL. Average NYHA class was 2.2.
Recruitment	Heart failure patients were stratified by presence of CHIP mutations for study inclusion. Patients were randomly chosen for from the heart failure clinic in at Frankfurt University Hospital for inclusion of CHIP screening. Informed consent was obtained from all patients. The study was approved by the local ethics review board and complies with the Declaration of Helsinki. Patients were eligible for inclusion into the study if they had stable chronic heart failure symptoms New York Heart Association (NYHA) classification of at least II, had a previous myocardial infarction at least 3 months before recruitment. Exclusion criteria were the presence of acutely decompensated heart failure with NYHA class IV, an acute ischemic event within 3 months prior to inclusion, a history of severe chronic diseases, documented cancer within the preceding 5 years, or unwillingness to participate. Heart failure patients with DNMT3A mutations were randomly assigned to the study group after inclusion criteria were met to minimize bias on the results.
Ethics oversight	The study was approved by the local ethics review board (Ethikkommission des Fachbereichs Medizin der Goethe-Universität) and complies with the Declaration of Helsinki.

Note that full information on the approval of the study protocol must also be provided in the manuscript.

Field-specific reporting

Please select the one below that is the best fit for your research. If you are not sure, read the appropriate sections before making your selection.

☒ Life sciences ☐ Behavioural & social sciences ☐ Ecological, evolutionary & environmental sciences

For a reference copy of the document with all sections, see nature.com/documents/nr-reporting-summary-flat.pdf

Life sciences study design

All studies must disclose on these points even when the disclosure is negative.

Sample size	Required sample sizes for experiments were estimated using power-calculation (p=0.8).
Data exclusions	Commonplace rules established, a priori, were utilized for exclusion of outliers (in vitro experiments) as determined by results from the Grubb's test (alpha=0.05).
Replication	For all experiments, the number of replicates and independent experiments is given in the respective figure legends.

Randomization

Patients in the study were randomly assigned for inclusion in the study after having been determined carriers of somatic DNMT3A mutations. Cells from healthy donors used for primary cell validation data were randomly assigned. Determination of wells to be used in silencing experiments were also applied in an unbiased manner.

Blinding

Automated processing of control and treated samples for immunofluorescence analysis was performed to minimize user bias. Flow cytometry of control and treated samples was performed with preset gating strategies to minimize influence of the operator. For detection of mutations, sample information was required.

Reporting for specific materials, systems and methods

We require information from authors about some types of materials, experimental systems and methods used in many studies. Here, indicate whether each material, system or method listed is relevant to your study. If you are not sure if a list item applies to your research, read the appropriate section before selecting a response.

Materials & experimental systems

n/a	Involved in the study	
☐	☒	Antibodies
☐	☒	Eukaryotic cell lines
☒	☐	Palaeontology and archaeology
☒	☐	Animals and other organisms
☐	☒	Clinical data
☒	☐	Dual use research of concern
☒	☐	Plants

Methods

n/a	Involved in the study	
☒	☐	ChIP-seq
☐	☒	Flow cytometry
☒	☐	MRI-based neuroimaging

Antibodies

Antibodies used

For flow cytometry, primary antibodies conjugated to a fluorophore were used. For immunofluorescence, targets were directly stained with fluorescently labeled primary antibodies and indirectly stained using specific primary antibodies in combination with fluorescently labeled secondary antibodies. For enrichment of monocytes and naive CD4 T cells from PBMCs, commercially available negative isolation kits were used. Unwanted targets were indirectly magnetically labeled with a cocktail of biotinylated primary antibodies and anti-biotin MicroBeads. Information on antibodies including clone, dilutions, vendor and catalogue number is provided in supplementary data and methods.

Flow cytometry
Mouse anti-human CD4-BV786, clone OKT4, #317442, BioLegend
Mouse anti-human CD45RA-AF700, clone HI100, #304120 , BioLegend
Mouse anti-human CD197(CCR7)-BV605, clone G043H7, #353224, BioLegend
Mouse anti-human CD4-FITC, clone RPA-T4, #300506, BioLegend
Mouse anti-human CD3-AF700 , clone OKT3, #317340, BioLegend
Mouse anti-human CD25-PE-Cy5, clone BC96, #302608 ,BioLegend
Mouse anti-human CD69-BUV395, clone FN50, #564364, BD
Mouse anti-human TNFA-APC-Cy7, clone Mab11, #502944, BioLegend
Mouse anti-human IFNG-BV605, clone 4SB3, #502536, BioLegend
Rat anti-human IL4-PE , clone MP4-25D2, #500810, BioLegend
Mouse anti-human IL17A-BV786, clone N49-653, #563745, BD
Mouse anti-human Tbet-BV421, clone 4B10, #644832, BioLegend
Mouse anti-human GATA3-BV711, clone L50-823, #565449, BD
Mouse anti-human RORC-PE-CF594, clone Q21-559, #567532, BD
Mouse anti-human CD56-FITC, clone MEM-188, #304604, BioLegend
Mouse anti-human CD3-BV786, clone OKT3, #317330, BioLegend
Mouse anti-human TNFA-APC-Cy7, clone Mab11, #502944, BioLegend
IFNG-BV711, clone 4SB3, #502540, BioLegend

Immunofluorescence
mouse anti-α -smooth muscle actin-Cy3, clone 1A4, #C6168, Sigma-Aldrich
rabbit anti-collagen type I, clone E8F4L, #72026, Cell Signaling
donkey anti-rabbit-647, #A-31573, Thermo Fisher Scientific

Magnetic-activated cell sorting:
Pan Monocyte Isolation Kit, human (#130-096-537, Miltenyi): Pan Monocyte Biotin-Antibody Cocktail containing biotin-conjugated monoclonal antibodies against antigens that are not expressed on human monocyte, Anti-Biotin MicroBeads (MicroBeads conjugated to monoclonal anti-biotin antibodies).
Naïve CD4+ T Cell Isolation Kit II, human (#130-094-131, Miltenyi): Biotin-Antibody Cocktail and MicroBead Cocktail.

Validation

Primary antibodies were validated by the manufacturer confirming specific labeling of target molecules and have been used in several peer-reviewed publications, as indicated in the links below.

https://www.biolegend.com/en-us/products/brilliant-violet-785-anti-human-cd4-antibody-7978

https://www.biolegend.com/en-us/products/alexa-fluor-700-anti-human-cd45ra-antibody-3421
https://www.biolegend.com/en-us/products/brilliant-violet-605-anti-human-cd197-ccr7-antibody-7674
https://www.biolegend.com/en-us/products/fitc-anti-human-cd4-antibody-825
https://www.biolegend.com/en-us/products/alexa-fluor-700-anti-human-cd3-antibody-9625
https://www.biolegend.com/en-us/products/pe-cyanine5-anti-human-cd25-antibody-617
https://www.bdbiosciences.com/en-us/products/reagents/flow-cytometry-reagents/research-reagents/single-color-antibodies-ruo/buv395-mouse-anti-human-cd69.564364
https://www.biolegend.com/en-us/products/apc-cyanine7-anti-human-tnf-alpha-antibody-9089
https://www.biolegend.com/en-us/products/brilliant-violet-605-anti-human-ifn-gamma-antibody-7677
https://www.biolegend.com/en-us/products/pe-anti-human-il-4-antibody-969
https://www.bdbiosciences.com/en-us/products/reagents/flow-cytometry-reagents/research-reagents/single-color-antibodies-ruo/bv786-mouse-anti-human-il-17a.563745
https://www.biolegend.com/en-us/products/brilliant-violet-421-anti-t-bet-antibody-7281
https://www.bdbiosciences.com/en-us/products/reagents/flow-cytometry-reagents/research-reagents/single-color-antibodies-ruo/bv711-mouse-anti-gata3.565449
https://www.bdbiosciences.com/en-us/products/reagents/flow-cytometry-reagents/research-reagents/single-color-antibodies-ruo/pe-cf594-mouse-anti-human-ror-t.567532
https://www.biolegend.com/en-us/products/fitc-anti-human-cd56-ncam-antibody-1604
https://www.biolegend.com/en-us/products/brilliant-violet-785-anti-human-cd3-antibody-7977
https://www.biolegend.com/en-us/products/apc-cyanine7-anti-human-tnf-alpha-antibody-9089
https://www.biolegend.com/en-us/products/brilliant-violet-711-anti-human-ifn-gamma-antibody-7949

https://www.sigmaaldrich.com/DE/de/product/sigma/c6198
https://www.cellsignal.com/products/primary-antibodies/col1a1-e8f4l-xp-rabbit-mab/72026
https://www.thermofisher.com/antibody/product/Donkey-anti-Rabbit-IgG-H-L-Highly-Cross-Adsorbed-Secondary-Antibody-Polyclonal/A-31573

https://www.miltenyibiotec.com/DE-en/products/pan-monocyte-isolation-kit-human.html#130-096-537
https://www.miltenyibiotec.com/DE-en/products/naive-cd4-t-cell-isolation-kit-ii-human.html#130-094-131

Eukaryotic cell lines

Policy information about cell lines and Sex and Gender in Research

Cell line source(s)

THP1 was purchased from the German Collection of Microorganisms and Cell Cultures (DSMZ, #ACC16), HUVECs were purchased from Lonza (#C2519A) and NKL was a kind gift from from Prof. Dr. Winfried Wells, Georg-Speyer Haus, Frankfurt am Main. NKL is currently not commercially available anymore but was available at ATCC in the past.
Human Cardiomyocyte Ventricular Primary Cells (HCM-VT) from Celprogen (#36044-15VT) primary human cardiac fibroblasts (HCF) from Promocell (#C-12375) are derived from primary donors. Monocytes and naive CD4 T cells were isolated from n=3 male and n=1 female and n=4 male and 2 female donors, respectively.

Authentication

Cell lines were not authenticated.

Mycoplasma contamination

Cell lines were regularly tested for mycoplasma by PCR using a commercially available kit (#11-1025, minerva biolabs) and were tested negative.

Commonly misidentified lines
(See ICLAC register)

No commonly misidentified cell lines were used in the study.

Clinical data

Policy information about clinical studies
All manuscripts should comply with the ICMJE guidelines for publication of clinical research and a completed CONSORT checklist must be included with all submissions.

Clinical trial registration

REPEAT trial (Repetitive Progenitor Cell Therapy in Advanced Chronic Heart Failure; NCT 01693042)

Study protocol

Assmus, B. et al. Improved outcome with repeated intracoronary injection of bone marrow-derived cells within a registry: rationale for the randomized outcome trial REPEAT. Eur. Heart J. 37, 1659–1666 (2016).

Data collection

Clinical data collection was conducted in accordance with the REPEAT trial study protocol and as noted in the methods sections of this article.

Outcomes

REPEAT trial aimed the comparison of the effects of single versus repeated intracoronary application of bone-marrow cells on 2-year mortality in patients with chronic post-infarction heart failure. While bone marrow samples of 19 consecutive REPEAT patients were investigated in this study (all at baseline, before the first intracoronary application of bone marrow cells, irrespectively of the study randomization), the outcome data from the REPEAT trial were not relevant for the current manuscript.

Flow Cytometry

Plots

Confirm that:

☒ The axis labels state the marker and fluorochrome used (e.g. CD4-FITC).

☒ The axis scales are clearly visible. Include numbers along axes only for bottom left plot of group (a 'group' is an analysis of identical markers).

☒ All plots are contour plots with outliers or pseudocolor plots.

☒ A numerical value for number of cells or percentage (with statistics) is provided.

Methodology

Sample preparation

Cultured primary cells (CD4 T cells differentiated from naive CD4 T cells isolated from peripheral blood of healthy donors) and cell lines (NKL and HUVEC) were analyzed by flow cytometry.
To detect intracellular cytokines, CD4 T cells were re-stimulated with PMA (50 ng/ml) and Ionomycin (1 µg/ml) in the presence of Brefeldin A (5µg/ml), (biolegend) for four hours. NKL cells were cultured in the presence of Brefeldin A for four hours. Around 1*10^6 cultured cells were washed with 2 ml Staining Buffer (ebiosciences) at 400 g for 5min. Surface molecules were stained in 100 µl Staining Buffer for 15 min followed by another washing step. For detection of intracellular targets, cells were fixed with an equal volume of IC Fixation Buffer (ebiosciences) for 20 min. Then, cells were permeabilized by washing cells with 2 ml Permeabilization Buffer (ebiosciences) followed by staining of targets in 100 µl Permeabilization buffer for 30 min. A final washing step with 2 ml Staining Buffer was carried out before flow cytometry analysis.

HUVECs were labeled with CFSE (CellTrace, Thermo) prior to co-culture with NK cells. In brief, after staining 1×10^6 cells with 5 µM CFSE in 1 ml PBS for 20 min at 37°C, remaining dye in the solution was quenched with five volumes of complete media for 5 min. Then, the cells were pelleted and resuspended in pre-warmed complete media. Cells were analyzed by flow cytometry after staining following a resting period of at least 10 min. Prior to co-culture with NK cells, HUVECs were rested overnight.
After co-culture with NK cells for four hours, cells were trypsinized, washed with Staining Buffer and stained with 5 µl 7-AAD (biolegend) for 15 min to identify dead cells. 7-AAD-positive HUVEC cells were determined by flow cytometry.

Preparation of peripheral blood mononuclear cells
Peripheral blood mononuclear cells (PBMCs) were isolated from Buffy Coats of healthy donors, obtained from the Blutspendedienst Frankfurt, by density gradient centrifugation. In brief, PBMC-enriched blood was diluted 1:3 with pre-warmed PBS supplemented with 0,5% BSA and 2 mM EDTA, and layered on top of human Pancoll (1.077 g/ml, Pan Biotech) followed by centrifugation at 400 g for 25 min with disabled break. The PBMC layer was collected, washed and centrifuged at 300 g for 10 min. Platelets were removed by two additional washing steps and centrifugation at 200 g for 10 min. Cell types of interest were enriched by Magnetic Activated Cell Sorting (MACS, Miltenyi). Monocytes and naïve CD4+ T cells were isolated using the human Pan Monocyte Isolation Kit and the Naïve CD4+ T Cell Isolation Kit II, respectively (all from Miltenyi), following the manufacturer's guidelines. The enrichment of desired cell types was confirmed by flow cytometry

Differentiation of naïve CD4+ T helper cells
After MACS isolation, naïve CD4+ T cells were seeded in 48-well plates (2×105 cells/well) and rested for three hours before transfection with 100 nM siRNA. The media was refreshed 16 hours post transfection followed by T cell activation and differentiation into T helper cell subsets. Naïve T cells were activated in in the presence of IL-2 (5 ng/ml) and CD3- and CD28 agonists (T Cell TransAct, Miltenyi) to generate Th0 cells. Th1, Th2 and Th17 cells were induced with IL-12 (20 ng/mL), IL-4 (20 ng/ml) and TGF-b (5 ng/mL) plus IL-6 (50 ng/mL), respectively. In addition, polarizing cytokines were replaced by supernatant from siRNA-transfected human primary macrophages used in 1:5 dilution for CD4+ T cell differentiation. Cells were differentiated for six days with a media exchange after three days. Supernatants of CD4+ T cells indirectly co-cultured with human macrophages were collected from day three to six of differentiation, diluted 1:2 with HCF media and used to stimulate HCF for 48 h.
Flow cytometry
Multicolor flow cytometry was performed to analyze surface markers in combination with intracellular transcription factors and secreted cytokines. The panels consisting of fluorochrome-conjugated antibodies in Extended Data Table 4 were applied. Potential spectral overlap in the emission of fluorochromes was compensated in advance with bulk PBMCs using unstained, single-stained, fluorescence-minus-one and complete stained controls.
For intracellular detection of secreted cytokines, differentiated CD4+ T cells were re-stimulated with PMA (50 ng/ml) and Ionomycin (1 µg/ml) in the presence of Brefeldin A (5µg/ml), (biolegend) for four hours. NK cells were treated with Brefeldin A for four hours prior to intracellular cytokine staining. Then, cells were processed for flow cytometry analysis as follows: After washing with 2 ml Staining Buffer (biolegend) and centrifugation at 400 g for 5 min, cell surface antigens were stained in 100 µl staining buffer. Afterwards, cells were washed with 2 ml Staining Buffer, centrifuged at 400 g for 5 min and fixed with an equal volume of IC Fixation Buffer (ebioscience) for 20 min. After washing the cells with 2 ml Permeabilization Buffer (ebioscience), intracellular targets were stained in 100 µl Permeabilization Buffer for 30 min. A final washing step with 2 ml Staining Buffer was carried out before flow cytometry analysis. Data was acquired with a LSR Fortessa X-20 Cell Analyzer and FACSDiva Software (BD Biosciences), fcs files were analyzed with FloJo Software (version 10.8.1).

CFSE staining
To distinguish human umbilical venous endothelial cells (HUVEC) from co-cultured NK cells, HUVECs were fluorescently labeled with CFSE (CellTrace, Thermo) according to the manufacturer's instruction. In brief, after staining 1×106 cells with 5 µM CFSE in 1 ml PBS for 20 min at 37°C, remaining dye in the solution was quenched with five volumes of complete media for 5 min. Then, the cells were pelleted and resuspended in pre-warmed complete media. Cells were analyzed by flow cytometry

after staining following a resting period of at least 10 min. Prior to co-culture with NK cells, HUVECs were rested overnight.

NK cell cytotoxicity assay
To study the cytotoxic capacity of NK cells on human umbilical venous endothelial cells (HUVEC), the NK cells were co-cultured with CFSE-labeled HUVEC target cells in a ratio of 1:1 for four hours. Then, cells were trypsinized, washed with Staining Buffer and stained with 5 µl 7-AAD (biolegend) for 15 min to identify dead cells. 7-AAD-positive HUVEC cells were determined by flow cytometry. The percentage of lysed HUVEC was calculated as follows: %CFSE+7-AAD+ HUVEC (co-culture) – %CFSE+7-AAD+ HUVEC (HUVEC only)

Instrument

Data was acquired with a LSR Fortessa X-20 Cell Analyzer

Software

BD FACSDiva Software 6.0 (BD Biosciences) for data acquisition, FlowJo 10.8.1 (BD Biosciences) for data analysis.

Cell population abundance

No cell sorting was performed.

Gating strategy

For all flow cytometry analysis live cells and single cells were gated. For analysis of CD4 T cells, activated CD4 T cells (CD4 +CD25+) were selected to identify targets of interest (Extended Data Figure 6b). For NKL cells, CD56, TNFA and IFNG were detected in live single cells (Extended Data Figure 7a). HUVECs lysed by NK cells were defined as 7-AAD-positive cells within CFSE-labeled HUVEC in single live and dead cells (Extended Data Figure 7b). Positive populations were identified based on the staining pattern of a positive control (bulk PBMCs) and the seperation of populations in contour plots.

☒ Tick this box to confirm that a figure exemplifying the gating strategy is provided in the Supplementary Information.
